# Statistical Reports on the Sickness, Mortality, and Invaliding among the Troops in Western Africa, St. Helena, the Cape of Good Hope, and the Mauritius

**Published:** 1840

**Authors:** 


### Medico-Chirurgical Review,

### No. LXV.

**[No. 25 of a Decennial Series.]**

APRIL 1, TO JULY 1, 1840.

### STATISTICAL REPORTS ON THE SICKNESS, MORTALITY, AND INVALIDING AMONG THE TROOPS IN WESTERN AFRICA, ST. HELENA, THE CAPE OF GOOD HOPE, AND THE MAURITIUS. Prepared from the Records of the Army Medical Department and War-Office Returns. 1840.

OUR readers will of course remember the particularity with which we noticed the official documents, relating to the health of our troops in the West Indies, and our North American colonies. Such statistical records have a wider bearing than the military force on which they have been founded, and affect not only our ideas of the salubrity of particular quarters of the globe, but the very principles of medicine itself. On this account, we seize the present opportunity of recurring to a subject of such interest to the whole profession, and of carrying on our notice of these statistical reports, which, much to its credit, are emanating from the War-Office.

Major Tulloch informs us, in his usual brief Introduction, that, in the present volume, are submitted the following Reports and relative Appendices:—

I. On the Sickness, Mortality, and Invaliding among the Troops serving on the Western Coast of Africa.

II. On the Sickness, Mortality, and Invaliding among those serving in St. Helena.

III. On the Sickness, Mortality, and Invaliding among those serving at the Cape of Good Hope.

IV. On the Sickness, Mortality, and Invaliding among those serving at the Mauritius.

With these would have been included a similar Report on the Health of the Troops in the Australian Colonies, but so many of the detachments there have been under the charge of civil practitioners, who do not furnish returns to the Army Medical Department, that the necessary information in regard to the prevailing diseases, cannot at present be procured. This defect, however, may yet be supplied; and in the meantime, the extreme salubrity of the climate may be estimated from the circumstance, that on the average of 20 years from 1817 to 1836 inclusive, the mortality did not exceed 14 per thousand of the force annually, whereof more than a fifth part arose from violent or accidental deaths, principally attributable to the nature of the duties on which the troops were employed. Thus the mortality from disease alone could have amounted to little more than one per cent. annually, being lower than in any other colony, except the eastern provinces of the Cape of Good Hope, to which the climate of Australia is in many respects similar.

We are glad to observe a warmer acknowledgment of medical services, in the construction of these Reports. Such courtesy is becoming as well as due. The gentlemen to whom Major Tulloch expresses his obligations are, Deputy Inspector General Marshall and Staff Assistant Surgeon Balfour.

### I. On the Sickness and Mortality among the Troops on the Western Coast of Africa.

The British settlements in Western Africa are scattered over a line of coast which, from St. Mary's on the Gambia to Accra, is nearly 1600 miles in extent, and consequently presents considerable diversity in climate, soil, surface, and geological structure. Unfortunately all spots agree in this—their deadly influence upon European life. The Report opens indeed with a significant comment on the pestilential character of these fatal shores—it mutilates even statistic documents.

"It is to be regretted that on this coast, where the baneful effects of climate on the European constitution exhibit themselves in their most concentrated form, and where it would have been of the utmost importance to trace the diseases of each station with the same minuteness as in previous Reports, the materials are neither so ample as those from other colonies, nor admit always of the same arrangement as has been hitherto adopted. The unceasing occupation of their professional duties in so unhealthy a climate left medical officers little time for making the proper distinction between the diseases of white and black troops; and their death has frequently prevented information from being obtained at those periods when the mortality was at its greatest height, and when an accurate statement of the particular circumstances under which it occurred would have been most interesting and useful."

Vos o quibus integer ævi

Sanguis, ait, solidæque suo stant robore vires;

Vos agitate fugam.

On the cession of Goree and Senegal to the French, the only stations occupied by the British troops on the coast were Sierra Leone, Gambia, and the Isles de Loss; but in 1821 the settlements of Cape Coast Castle and Accra, with their dependencies, having been transferred to the British Government by the African Company, were formed into another Command, of which it will be necessary to investigate the sickness and mortality separately.

### a. *Sierra Leone Command.*

Major Tulloch offers a brief account of the localities of the stations of Sierra Leone, the Isles de Loss, and Gambia.

The peninsula of Sierra Leone comprehends a tract of land extending about 18 miles from north to south, and 12 from east to west, consisting principally of a range of conical mountains from 2000 to 3000 feet in height, surrounded by a belt of level ground from one to five miles in breadth, to which have recently been added the Banana Islands and some minor acquisitions of territory.

From the north to the south-east the whole country adjoining the peninsula is intersected by numerous creeks and rivers, which, overflowing during the rainy season, form extensive swamps in every direction. Indeed, it may be stated generally, that the whole coast from Senegal to Sierra Leone, a distance of above 700 miles, is exceedingly low, being not more than a few feet above the ocean; that the rivers with which it is intersected are sluggish in their course, and flooded during the rains, when the mud they deposit, and the moisture they supply, give birth to an interminable wilderness of forest and brushwood, among which lies rotting the decayed vegetation of many centuries.

From the noxious agencies likely to be generated in such a tract, the peninsula of Sierra Leone, however, is sheltered by the mountain ranges which form the boundary in that direction, while on the south it is washed by the Atlantic, and on the north by an estuary terminating in the Sierra Leone river, so that it seems protected by nature from all extraneous sources of disease, except such as may originate on the opposite side of the river, called the Bulam shore; and, so far as can be ascertained, there is nothing within the limit of the colony itself, likely to induce that sickness which has proved so fatal to every class of the population.

From the census of 1838, it appears that the population of Sierra Leone then consisted of about 42,000 persons—of whom there were:—whites, 83 males, 19 females; blacks, 21,559 males, 18,381 females; aliens, 1,681 males and females.

*Free Town*, the capital, stands on the south bank of the Sierra Leone river, about five miles from the sea. At the distance of about a mile it is surrounded on the west, south, and east, by a semi-circular range of lofty hills, clothed to the summit with high trees and thick underwood, and to which there is a gradual ascent from the river. This position gives it a very pleasing aspect, by no means calculated to impress an idea of insalubrity, and as the soil is gravelly, and has a gentle slope, the rain is speedily absorbed or drained off even during the most inclement periods of the wet season. The houses are mostly built of wood and disposed in broad and regular streets, in many places yet uncleared of the brush and underwood with which the site was originally covered. There is little cultivation in the immediate vicinity of the town, the soil not possessing great capabilities for agriculture; but considerable progress has been made at some of the villages in the interior, where it is better adapted for that purpose.

The town is elevated about fifty feet above the river, the banks of which were formerly covered with a dense barrier of mangrove bushes. This, having been supposed a fertile source of disease, was cleared to a considerable extent, without however effecting any obvious improvement in salubrity. The distance from the Bulam shore opposite is about seven miles; the soil on that shore is a ferruginous clayey loam, which is apt to form marshes during the rainy season, owing to the surface being almost level. In the earlier years to which this Report refers it was covered with thick brush and underwood, and supposed in some instances to have given rise to the unhealthiness of the colony; but though cultivation has since made considerable progress in that quarter, and the face of the country is rapidly improving, no corresponding amelioration has been permanently manifested in salubrity.

The *Isles de Loss* lie about 60 miles north-west of Sierra Leone. Only three are habitable. The nearest is three, and the most distant eight, miles from the main land. A few disbanded black soldiers and two or three Europeans, who have established a factory there, form, with the detachment, the only inhabitants. Crawford's Island, on which the troops are stationed, is the centre of the group, and is described as entirely composed of granite, elevated 250 feet above the level of the sea, and about a mile and a half in length, though scarcely 100 yards in breadth. It is entirely exempt from that exuberance of vegetation which prevails on the main land, and in no part of it, nor in any of the other islands, are either pools or marshes to be found.

The *Island of St. Mary's*, the principal settlement on the Gambia, is about five miles in length and one in breadth, and consists merely of a sand-bank, formed by the confluence of the tides at the mouth of the river. It is consequently in many places under high-water mark, which renders it, during the rainy-season, one complete marsh. The soil is too light to be well fitted for agriculture, but, where intersected by creeks and aided by the alluvial deposits brought down by the river, gives birth to dense masses of mangroves, underwood, and every species of rank vegetation, which, particularly during the hot season, create most offensive effluvia throughout the whole island.

The *Town of Bathurst* is built on the east side of the island, along the beach, upon a ridge of sand somewhat elevated above the level of the sea. Efforts have of late been made, by the construction of dykes and drains, to render the town less swampy during the rainy season, and thus improve its salubrity, but without much success. In 1826 the inhabitants, exclusive of the military, amounted to 1867, of whom only 20 were Europeans.

Such is the *locale* of the Sierra Leone Command. The main characteristic of the *climate* is extreme humidity, a circumstance which is sufficiently attested by the fact of more rain having fallen in two successive days, the 22nd and 23rd of August, 1828, than in Britain throughout the whole year. The quantity of rain at the Gambia is not so great as at Sierra Leone.

The *Temperature*, compared with that of similar latitudes exhibits no very marked peculiarity. With the exception of Sierra Leone, where the diurnal range of the thermometer rarely exceeds 10°, sudden transitions from heat to cold, with dense and chilling fogs for many months of the year, form the general characteristic of the whole west coast of Africa, particularly at the Gambia, where the thermometer has sometimes fallen as low as 62° in the morning, during October, November, and December, and then risen to 80° in the course of a few hours. These sudden changes of temperature, observes Major Tulloch, are by no means uncommon in tropical climates. The thermometer, for example, at Seringapatam, has been observed at 54° in the morning, accompanied by cold dense fogs, and as high as 92° by mid-day, being a much wider range than on this coast, without any great degree of mortality being thereby induced.

The Peninsula of Sierra Leone, though beyond the trade winds, enjoys a regular succession of sea and land breezes, the former commencing about nine o'clock A.M. from the West-North-West, always cool and pleasant, the latter setting in about the same hour in the evening, from the East and South-East, but generally heated, and laden with humid exhalations from the low and swampy ground over which it passes. The interval between the dying away of one breeze and the springing up of another is always hot and oppressive.

At the Isles de Loss and Gambia the land and sea breezes are generally from the north-west and south-west. All along the coast there prevails, during the months of December, January, and February, a dry, parching, easterly wind, termed the Harmattan; but, except on catarrhal and pulmonary affections, its influence seems rather favourable than prejudicial to health.

The *wet season* extends, at Sierra Leone and the Isles de Loss, from May to November, and at the Gambia from June to September or October, and is always ushered in and carried off by tornados. Nothing can exceed the gloominess of the weather during this period: the hills are wrapped in impenetrable fogs, and the rain falls in such torrents as to preclude that exercise and amusement which are so necessary to invigorate the body and give energy to the mind. At this period, the diseases which prove so fatal on the coast have generally made their appearance, though there have been so many exceptions, that they can scarcely be said to belong peculiarly to any season.

During most of the period under review, the force consisted of a colonial corps of white and black troops, the former unfortunately of a class the least fitted to contend with such a climate, being principally soldiers allowed to volunteer their services as a commutation of punishment, and whose vices and intemperance, no doubt, in many instances facilitated the inroads of disease.

Till 1817 the number of white and black soldiers was nearly equal, but in that year most of the former were removed to the frontier settlements of the Cape of Good Hope; and in 1819 the whole corps was disbanded and replaced by the 2nd West India Regiment, composed, with the exception of a few serjeants, entirely of negroes. In 1823, however, a war having broke out with the Ashantees, the white soldiers formerly disbanded at the Cape of Good Hope were hastily re-embodied and sent to the defence of Cape Coast Castle; the survivors of these were subsequently transferred to the Sierra Leone Command, and, with several drafts of commuted punishment men from England, formed into the Royal African Colonial Corps, which thus again consisted of Europeans of the most degraded class. Death, however, thinned the ranks with such rapidity that an attempt had to be made, in 1825, to keep up the force by voluntary enlistment; above 100 recruits were in this manner raised and sent from Britain, but though their character and conduct appear to have been unexceptionable, they soon shared the same fate as their depraved comrades. The impossibility of maintaining white troops in such a climate being thus demonstrated, the garrison has, since the end of 1829, consisted entirely of blacks, with the exception of a few European serjeants.

The *Barracks and Hospitals*, prior to 1836, appear to have been infamous. What must have been the horror, how natural the mortality, during a Sierra Leone wet season, when even many of the officers could obtain no better accommodation than rude huts, incapable of affording shelter from the inclemency of the rains, and in which it was not uncommon to find the husband, wife, and children lying in the last extremity of fever in the same room.

But in 1826, good barracks were built at Sierra Leone, on the summit of a conical hill 400 feet high, nearly in the centre of the elevated range which surrounds Free Town. The barracks at the Gambia is not so good, being near the lowest ground; and that at M'Carthy's Island is worse. The *rations* are now unexceptionable, but, previously to 1827, that was far from the case.

*Mortality.*—From a table furnished by Major Tulloch, it appears that, during so long a period as 18 years, the admissions have averaged 2,978, and the deaths 483 per thousand of the strength; in other words, every soldier was thrice under medical treatment, and nearly half the force perished annually; indeed, in 1825 and again in 1826, when the mortality was at its height, three-fourths of the force were cut off. Yet this estimate excludes the deaths from accidents, violence, &c.

A considerable portion of the deaths in 1825 and 1826 took place at the Gambia, which proved the grave of almost every European sent there. Had the mortality of each station been kept distinct, that of the European troops at Sierra Leone would not probably have exceeded 350 per thousand, or rather more than a third of the garrison annually.

There can be no doubt that the casualties of 1825 and 1826 raised the mortality in this command considerably above the usual average, but in almost every year it has proved exceedingly inimical to Europeans. From information supplied by the Report of the Sierra Leone Commissioners, it appears that during the nine years antecedent to 1819, the mortality of the white troops on this coast averaged about a fourth part of their number annually, and even so far back as 1792, we find that the loss of the soldiers and white colonists sent out by the Sierra Leone Company was in the same proportion.

However much the vice and intemperance, not only of the troops but of the other classes of the white population, may have aggravated the mortality, a more regulated life and purer morals brought no safety with them. For, among the missionaries, we find that:—

| | |
|---|---|
| Of 89 who arrived between March 1804 and August 1825, all men in the prime of life, there died | 54 |
| Returned to England in bad health | 14 |
| .. .. in good health | 7 |
| Remained on the coast | 14 |
| Total | 89 |

From a table given by Major Tulloch, it is evident that the principal source of sickness and mortality has been fever, by which every white soldier has on an average been attacked once in nine months, and more than two-fifths of the force have been cut off annually. The remittent type has been both the most prevalent, and infinitely the most fatal, out of 2,600 admissions, 1,601 being from this, and 739 deaths out of 756.

The mortality, not including the most unhealthy years, would appear to have been about 17 per cent. annually. But in common with all tropical climates, that of Western Africa exhibits remarkable variations in salubrity in different years, without any obvious cause to induce such a change ; between 1830 and 1836, for instance, the Colony enjoyed a succession of such healthy years as to induce the supposition that the causes which had rendered it so long a terror to Europeans were never likely to come again into operation ; but the fatal epidemics of 1837 and 1838 dispelled this pleasing delusion, and showed how little reliance could be placed on what were merely the vicissitudes characteristic of a treacherous climate.

" So generally prevalent is remittent fever, either in its aggravated or milder forms, on this coast, that till of late years scarcely any European ever passed twelve months without an attack ; the most regular habits and the best constitution afforded no protection, nor did a residence on the coast, however long, secure any immunity. It was observed in 1825, and a few of the following years, that the appearance of ulcers, which were very common among those soldiers who indulged in intemperance, seemed to act as a safeguard against remittent fever so long as they continued open and discharged freely, but whenever they ceased to do so, that disease assailed the patient and generally proved fatal. More recently, in 1837, it was also observed that in many of the worst cases, an exudation of blood took place from the tongue, gums, nose, and anus, and, that whenever leeches were applied, the tendency to hæmorrhage was so great as to render it almost impossible to stop the effusion. Except in these respects there appears to have been little difference in the character of the disease from that which generally marks the course of yellow fever of the worst type in other Colonies. On this point, it may be proper, however, to remark, that, between 1824 and 1829, the black vomit is not mentioned in any of the Reports as one of the characteristics of the disease. This may perhaps induce a doubt in the minds of those who attach importance to that symptom, as to whether the disease in these years was genuine yellow fever, or merely the endemial remittent of the country ; but as the latter is comparatively of a mild character, the cases could scarcely have been of that type, when in general one-half, and in some instances three-fourths, perished, of all those attacked." 9.

The disease, in 1835 and 1836, was more fatal at the Gambia, where three-fourths of those attacked died, than at Sierra Leone, where the deaths were about one-half. And it has been already observed that it has fluctuated remarkably in different years, both in prevalence and severity. In 1830 and the six subsequent years, it almost disappeared, while, in 1837 and 1838, it prevailed with all its ancient virulence. The following statements prove how little we know of the real causes which light up this pestilence into activity.

" This disease has, in most years, appeared and raged with the greatest violence during the height of the rainy season, when vegetation was most vigorous and healthy, and the low grounds, being completely flooded, were in the state supposed least favourable to the extrication of miasma ; it has also been observed to diminish both in prevalence and severity as the rains moderated and the marshes began to dry, but to this rule we have to record several exceptions ; —for instance, in 1823, 1829, 1837, and 1838, the disease appeared in its most malignant form in the months of February and March, during what had generally been termed the dry or healthy season, and on each of these occasions its violence declined as the rainy season advanced, and the earth became completely saturated with moisture, being directly the reverse of what had been observed in other years.

In most instances, too, the prevalence and fatal character of the disease have been commensurate with the quantity of rain; but here again the exceptions are so numerous as to strike completely at the root of all theories tending to establish that the former is a necessary result of the latter, for in 1812, 1823, and 1829, less rain fell than usual, yet fever was exceedingly prevalent, and though from 1830 to 1836 the Colony was almost free from fever, yet, with the exception of 1832, the quantity of rain in each year was about the usual average.

Attempts have also been made to connect the appearance of this disease with the circumstance of the rains commencing earlier or later, or being heavier or lighter at the commencement than usual, but without any satisfactory result, the exceptions being always too numerous to admit of any positive conclusions. The range of temperature, the fluctuations of the barometer, the direction and prevalence of the winds, have also been carefully observed, but do not seem in any way connected with it. The latest, as well as the earliest observations, all tend to show that the circumstances which call it into operation at one season or in one year more than another, have hitherto received no satisfactory explanation. In 1823, when the fever broke out during what was deemed the healthy season, and when none of the usual theories would account for its appearance, it was supposed by some of the medical officers to have originated in noxious miasma, wafted from the Bulam shore; but, so far as could be learnt, there was nothing in the character of that shore more likely to have produced the disease in 1823, than in other years which were healthy. And its subsequent appearance in 1837 and 1838 at the same season, when there was nothing to induce such a supposition as to its origin, certainly appears to indicate that there could have been but little foundation for this theory."

The elevated spots are not exempt from the fever. The barrack, though at the height of 400 feet, has frequently been the scene of greater mortality than the lowest situations in the town; and recently, when a body of seamen was removed to the village of Wilberforce, at an elevation of 500 feet, with the view of keeping them free from fever, they suffered even to a greater extent than on board the shipping in the harbour.

The common continued fever being ten times more fatal than in other colonies, probably partook of the remittent type.

*Diseases of the Lungs.*—The climate of this command seems rather favourable to diseases of this class, the admissions, compared with those which occur among an equal number of troops in the United Kingdom, being as 56 to 148, and the deaths as 4·9 to 7·7. The same feature was manifest in the diseases of the black troops, who are very prone to pulmonic affections in other colonies. The most marked exemption in this class is from inflammation of the lungs, by which only 8 per thousand have been attacked annually, though the usual proportion in most other colonies is from 30 to 40 per thousand. Consumption has also been comparatively rare. But really it is hard to conceive how a man *is* to die of consumption in these parts—he literally has not time for it.

Catarrhal affections, particularly those of an acute nature, were also extremely rare during the period when these white troops were employed; but of late an epidemic, resembling influenza, has prevailed annually in the command. It pervades simultaneously very extensive districts; generally commencing about the height of the dry season, when a keen, parching, easterly wind, termed the harmattan, blows. The native inhabitants have suffered more than the troops, but it has rarely proved serious or fatal, except to aged persons, or those debilitated by disease.

It is certainly singular how these epidemic catarrhs would appear to have increased, of late years, throughout the world. Abstractedly speaking, there is nothing to excite surprise in the occurrence of catarrhal affections in a climate like that of Sierra Leone. It is the increase of them that is odd.

*Diseases of the Liver.*—This class of diseases has been nearly four times more prevalent and fatal among the white troops in the command, than in any other colony of which the statistical details have yet been investigated.

*Diseases of the Stomach and Bowels.*—A table shews that more than half the troops have been under treatment, and that the deaths from these diseases have averaged 41 per thousand of the force annually. As usual, dysentery was the principal source of mortality, and of so aggravated a character were the acute cases, that nearly two-fifths of them proved fatal, a degree of intensity never surpassed elsewhere. There was a marked diminution of mortality from this cause, after the alteration of rations, and the liberal issue of fresh meat. The deaths were reduced to a tenth of their former number. Similar results have attended the recent increase in the issue of fresh meat to the troops in the West Indies, a circumstance which seems to warrant the adoption of a similar remedy in other colonies, whenever there is reason to believe that the character of these diseases has been influenced by a similar cause.

*Diseases of the Brain.*—This class of diseases has proved considerably more fatal than in any of the other Colonies, and, with the single exception of the Windward and Leeward Command, has also been more prevalent. Nor need we wonder at this when we consider the character and habits of the men.

*Dropsies.*—As these diseases are so often induced by attacks of remittent or intermittent fever, they have been much more prevalent and fatal than in other Commands.

*Venereal Affections.*—The troops at Sierra Leone enjoy a similar exemption to those in the Windward and Leeward Command, though not to so great an extent. This is principally manifested in syphilitic affections, of which only four primary cases occurred among the whites, and but three among the black troops in the whole course of eighteen years; yet no sanatory regulations appear to have been adopted.

*Abscesses and Ulcers*—extremely prevalent. Many were of a very serious nature, being apt to slough, and frequently causing such loss of substance as to create a permanent disability, and in some instances to render amputation necessary.

*Diseased Spleen* was exceedingly rife.

*Corporal Punishment* was both common and carried to great lengths. "In seven instances death ensued before the patient came out of hospital. Six of these, however, occurred at the Gambia, where, as the majority of each detachment generally perished within three months, it is probable their fate may only have been accelerated—not induced—by the punishment." What a comment upon climate! How demoralising is pestilence! The Reporter next examines the insalubrity of the different stations in detail. He is anxious to let Sierra Leone have only the responsibility of its own deaths—to give, in fact, the devil his due. First, then, of—

*Gambia.*—Could any idea bordering on the ludicrous attach to so melancholy a subject, the manner in which successive relays of troops were *dispatched* to or *in* this station would give birth to it. We know not how a sad tale of disease and death could be told more tersely and more affectingly than in the following statistic fashion.

The first detachment of white troops arrived in the latter end of May 1825, just as the rains commenced. It consisted of 108 men, being all for whom accommodation could be procured. Between that date and the 21st September of the same year, the casualties among them were as follows:

| | |
|---|---|
| Died of remittent fever .. .. .. .. .. | 74 |
| Of other diseases.. .. .. .. .. .. .. | 13 |
| Total died .. .. .. .. | 87 |
| Remained alive at the end of 4 months .. | 21 |

Owing to the want of sufficient accommodation on shore, another detachment of about 91 men was, during this period, kept at sea on board the *Surrey* transport, and while there, did not lose a man; but when, towards the end of September, room was provided for them in the barrack, by the death of four-fifths of their comrades, they were landed, and made up the force to about 112, of whom, between that period and the 21st December,

| | |
|---|---|
| There died of fever .. .. .. .. .. .. | 61 |
| Of other diseases, including 6 from fever following punishment .. .. .. | 12 |
| Total died .. .. .. .. | 73 |
| Remained alive on 21st December .. .. | 39 |

The force having now been reduced by deaths to 39, most of whom were in the last stage of disease, another body of about 200 Europeans was sent to supply their place, and that too, as before, at the commencement of the rainy season. It does not appear whether they were all landed at once, or a portion of them kept on shipboard, as on the previous occasion, for want of accommodation; but ere three months had elapsed, half the number were in their graves. The deaths reported from 21st June to 21st September of that year, were—

| | |
|---|---|
| Of fever.. .. .. .. .. .. .. .. .. .. | 85 |
| Other causes.. .. .. .. .. .. .. .. .. | 13 |
| Total in 3 months .. | 98 |

The strength appears to have been by this time reduced to 108, of whom there died from the 21st September to 21st December—

| | |
|---|---|
| Of fever.. .. .. .. .. .. .. .. .. | 14 |
| Of other diseases.. .. .. .. .. .. .. | 4 |
| Total.. .. .. .. .. | 18 |

Thirty-three of the survivors who were suffering under chronic affections of the liver, spleen, and other viscera, were then removed to head-quarters; of the remainder only one died, between January and July of the following year, when all the white troops were withdrawn from the station.

During the whole of this dreadful mortality, a detachment of from 40 to 50 black soldiers of the 2nd West India Regiment only lost one man, and had seldom any in hospital.

Fever accounts for all. Only about a fifth of those attacked came out of hospital alive. And those who did recover had a constitution shattered for life. The men were, naturally, reckless. Constant intoxication drowned the sense of danger, and they were drunk to-day, for to-morrow they knew that they would die. Perhaps reason was more on their side than it generally is in such a case. For observe what happened at—

*The Isles de Loss.*—Deceived by the supposed advantages of its situation, General Turner selected the centre island as a suitable station for a detachment of recruits voluntarily enlisted at Chatham, whom it was deemed advisable to separate from the commuted punishment men, to prevent their initiation into habits of intemperance and debauchery. They are described as being generally men of good character, exemplary conduct, and with little inclination to inebriety, in which, however, had they been ever so much inclined, they had no opportunity of indulging, as spirits could not be procured in the island.

The detachment arrived at the Isles de Loss on the 23rd of February, 1825, and consisted of 103 men. In 18 months, 62 were dead, and 21 were invalided to England. There remained then, of the original force 20, who were withdrawn at the end of the year, scarcely any being fit for duty! This, we may presume, will be the last experiment on the possibility of keeping white troops alive on the Western Coast of Africa.

It appears that, in 1825 and 1826, the relative mortality of each station was as follows:

| | | |
|---|---|---|
| Sierra Leone .... | 650 | per thousand of force employed, |
| Isle de Loss ...... | 600 | ,, ,, |
| Gambia.......... | 1,500 | ,, ,, |

and making allowance for the mortality at the subordinate stations, that at Sierra Leone alone, taken on the average of the whole 18 years, will be reduced to about one-third annually of the white troops employed.

*Black Troops.*—These have generally been recruited from the slaves captured by our cruizers, and liberated at Sierra Leone. Yet, on his native continent, supplied with all the necessaries of life, the negro soldier has not escaped disease. The mortality during the last 18 years has averaged about 30 per thousand exclusive of sudden and accidental deaths not stated in the medical returns, and which would probably have increased the ratio to 32 per thousand annually. At the lowest computation, it has been twice, and in some instances more than thrice as high as among other troops serving in their native country, though from the recent formation of the African Corps most of them are considerably under the average age of soldiers. The ratio will be found exactly the same as among the black troops employed in Jamaica and Honduras; and though less than in the Bahamas and Windward and Leeward Command, in the proportion of 32 to 41, yet as a very large proportion of the force there was of advanced ages, while in the African corps scarcely any soldier exceeded 25, the former ratio may be held to correspond very nearly with the latter; consequently, on his own native coast, even with all the advantages enjoyed by the British soldier, the negro exhibits a liability to mortality for which it is extremely difficult to account. Perhaps his previous treatment in the slaver had deteriorated his constitution, yet, making every allowance for this, there is abundant evidence that the climate is unfavourable even to the negro race.

In the years 1818 and 1819, as previously stated, 1,222 black soldiers were discharged at Sierra Leone, not in consequence of age and infirmities, but owing to the reduction of their corps. They are described as having been mostly in the prime of life, and of quiet, sober, industrious habits; each received a pension of from 6d. to 8d. per day, which, with an allotment of land and the produce of their daily labour, placed them in comparative affluence, yet, under all these favourable circumstances, at the census of April 1826 they were reduced by death to 949, making a total mortality of 273 in eight years, or in the ratio of 31 per thousand annually, which corresponds to the mortality among pensioners in this country about the age of 55, whereas these men could not have averaged above 40. Even among the negro colonists the same mortality obtains, and we suppose that the climate must be regarded as a villainous one to the whole human race. It is not however, from fever that the negro soldier suffers: from this the attacks have been fewer, and the deaths have not materially exceeded the proportion among an equal number of white troops in the United Kingdom or other temperate climates. The black troops suffer much more from fever in the West Indies. Small-pox has proved very destructive to them. Dracunculus infests them greatly. They do not suffer from diseases of the lungs half so much as in other colonies.

### B. *Cape Coast Command.*

In this Command were comprised the stations along what is termed the Gold Coast, extending from Dixcove to Accra, a distance of 150 miles. Since the end of 1828, however, when Fernando Po was selected as a settlement, none of them have been garrisoned by British troops. The settlements have little ground attached to them beyond the forts and gun-shot range.

*Cape Coast Castle*, the principal station, lies in Lat. 5° 6′ N. Long. 1° 10′ W. nearly 1000 miles to the East and South of Sierra Leone, and is built on a rock about 50 feet high, jutting into the sea, so that its walls are washed by the surf which rolls impetuously along the coast, and adds to the natural moistness of the atmosphere. The fort or castle is of a quadrangular shape, with bastions at each angle; the barracks are described as comfortable, well ventilated, and affording accommodation for 16 officers and 200 men; but having been occupied in 1825 and 1826 by considerably more than that number, they were extremely crowded. The castle is a place of little strength, the walls being out of repair, and commanded in every direction by the adjacent heights. The water for the garrison is obtained from tanks, in which the rain from the buildings is collected.

This part of the coast is not mountainous, but, at the distance of a quarter of a mile from the shore, a succession of small hills rise to the height of 150 or 200 feet, which, with the intervening valleys, are covered with forest trees and luxuriant vegetation throughout the year. The soil on the heights is of a siliceous nature, and in the valleys consists of a rich alluvial deposit. There is a salt pond about a mile distant, but no swamps or marshes exist in the vicinity, nor any river nearer than 5 or 6 miles.

Immediately under the walls of the fort is a native town containing about 5000 inhabitants; it is very much crowded, badly built, and, from its extreme filthiness, is supposed in some degree to have contributed to the unhealthiness of the garrison.

Of Dixcove and Annamaboe, we need not speak.

*Accra*, sixty miles east of Cape Coast Castle. In its vicinity, the country is open and level, free from bush or underwood to a considerable distance from the sea, and presenting the appearance of an extensive park. A small and rudely constructed fort protects the settlement, to which a detachment was generally furnished from Cape Coast Castle. The soil in the vicinity is sandy and mixed with vegetable mould; there are no swamps, no river nearer than eight miles, nor any of those noxious agencies which are generally supposed to contribute to insalubrity; yet, so fatal did the station prove, that in 1827 it became necessary to withdraw the troops and leave it in possession of one of the resident merchants, with local military rank, who now hires a few natives for its defence.

The *climate* of this coast is similar to that of Sierra Leone, extreme humidity being its principal characteristic.

While the African Company held the forts in this Command, they maintained a force of 120 natives for their protection, under European officers, who also followed the occupation of traders. When the British government took possession, these troops were re-enlisted into the service, and increased to 200 by native recruits. Owing to the Ashantee war, in 1823, a part of the Royal African Corps, formerly disbanded at the Cape of Good Hope, was re-embodied and augmented by drafts of "commuted-punishment men" from Europe, so that here, as at Sierra Leone, the greater portion of the white troops consisted of the most degraded class of soldiers. So fatal did the climate prove to them, that in the end of 1826 the few who survived were withdrawn, and their place supplied by two companies of natives. On the abandonment of Accra and Annamaboe, these were reduced to one, which, in October 1828, was transferred to Fernando Po, and no regular troops have since been employed on the coast. The rations of the white troops comprised no fresh meat, and few vegetables.

On the average of 1823, 4, 5, 6, two-thirds of the white troops died annually, and so great was the mortality in 1824, that the deaths nearly equalled the mean strength of the garrison. It has on this account been necessary to enumerate the strength in each quarter, as the troops were cut off with such rapidity, that few lived to complete one year in the Command. The following is a pithy history of some of the first detachments, in a medical report dated December, 1824.

"Out of the first detachment of European troops, which arrived in April 1822, under the command of Captain Donald, only *one* survives. Out of the second detachment of 129, which arrived in October 1823, under the command of Captain L'Estrange, only a few remain, and their constitutions are so destroyed by repeated attacks of intermittent and remittent fevers with dysentery, that their existence cannot be long. Out of a third detachment of 131 disembarked here on the 12th of March 1824, under the command of Lieutenant M'Combie, from the Cape of Good Hope, the majority died, a few months after landing, from remittent fever and dysentery depending on diseased liver. Out of the fourth detachment, which arrived on the 20th March last in the brig Anne from England, consisting of 33 men (chiefly non-commissioned officers) under the command of Lieutenant Mollan, only *six* are now alive. Out of the fifth detachment, which arrived from England in H. M. ship Thetis on the 4th of July last, and consisting of 101 men, 45 have already died. Out of the sixth detachment of 11 Artillerymen, who arrived here on the 17th of July 1824, from England, only one is dead, but I apprehend that ere long most of them will be on the sick list."

It was just the same at Accra as elsewhere, and with civilians as the soldiery. Out of 21 merchants who arrived in 1822, four only survived in 1825; out of 77 officers who arrived at various times between 1822 and 1827, no less than 37 died before quitting the Command; while of 42 women and 67 children landed with a detachment in October 1823, 29 women and 41 children perished in less than 15 months.

On referring to earlier years, before the coast was garrisoned by our troops, we find that of 40 Europeans in the service of the African Company, 15 died and 4 returned to England in bad health, in the years 1819, 1820, and 1821; and according to some Returns extending back to the year 1812, the loss of life appears to have been much the same, so that, in the healthiest years and under the most favourable circumstances, the mortality may be rated at from 12 to 13 per cent. annually.

Fever was much the same as in the Sierra Leone Command—diseases of the bowels much more fatal. The salt provisions must have contributed to this.

*Fernando Po.*—This island was selected as a military station from its supposed salubrity, and the facility it afforded for the protection of trade, and the location of slaves captured by our cruizers. It lies in the Bight of Biafra, 500 miles to the East of Cape Coast Castle, and separated, by a strait 20 miles in breadth, from the nearest point of the African Continent. It is about 120 miles in circumference, and, like that part of the mainland adjacent, is exceedingly mountainous, Clarence Peak, the most elevated point, attaining the height of several thousand feet. The southern extremity is also intersected by several steep mountains, varying from 1000 to 3000 feet, which, with the intervening valleys, are covered with dense forests of large and valuable timber, and watered by numerous rivulets.

Clarence Town, the principal settlement, lies in lat. 3° 53′ N., long. 7° 40′ E., and is built close to the sea upon an elevated plain from 100 to 200 feet in height, embracing two small peninsulas, Point William and Point Adelaide, with a semi-circular space extending about a mile in length, and forming a cove well adapted for shipping. All the ground in the immediate vicinity is covered with forest trees and jungle, except to the extent of about six square miles, which was partially cleared on the formation of the settlement. The soil, which is generally argillaceous, resting on a bed of freestone, gives proofs of abundant fertility when cultivated. The water, both of spring and brook, is of the best quality, and there are no marshes in the vicinity, the hilly nature of the ground not admitting of their formation.

The seasons, &c. are much as at the other stations on the coast. The land breeze is deficient. The only information obtained is that from Captain Vidall, R.N. given before the Parliamentary Commissioners. Of 40 European mechanics, sent out under his superintendence to form the settlement in 1827 and 1828, four died in the course of a few months. In 1830 the number of whites, including officers, was reduced to 16 or 18. Colonel Edward Nicholls then arrived, bringing with him 31 Europeans, principally mechanics, of whom 19 died in that year, besides 4 out of 12 shipwrights who came during the previous year to the settlement.

Fever and the worst ulcers prevail. In 1834, the detachment of black troops was withdrawn, and the island is no longer occupied as a military station.

On the whole, it seems that, 1,685 troops went to Western Africa. Of these there died, 1,298, and there were invalided the remainder, viz. 387 !

*Deductions from the preceding Report.*—These occupy a section.

Major Tulloch observes, that the opportunities for investigating the causes of remittent fever would seem to be most abundant, and most likely to be productive of results on the Western Coast of Africa, while the zeal with which that investigation was commenced, and has been carried on also, seemed to promise success. Yet all who have studied the subject have confessed both their ignorance and disappointment.

"The hypothesis that this fever originates from the miasma of marshes in the immediate vicinity of the station, as elsewhere it has been supposed to do, is directly opposed to the fact of the Isles de Loss, Accra, and the peninsula of Sierra Leone itself, being so subject to it, though all are, in a certain degree, remote from the operation of any such agency. If it be referred to similar exhalations wafted to the distance of several miles, how is its prevalence to be accounted for at Fernando Po, a mountainous region and bordering on a mainland still more so, and where, so far as can be ascertained, no such agency is in operation ? Instances of the disease having raged with the same violence on the rocky Isles de Loss and the sandy wastes of Senegal as in those parts of the coast where vegetation is most dense, preclude the likelihood of it originating in a superabundance of that agency. In every description of situation along the coast has this scourge of Europeans been found to prevail. The low swampy Gambia, the barren Isles de Loss, the beautifully diversified features of Sierra Leone, the open and park-like territory around Accra, the low jungle-covered hills of Cape Coast Castle, and the rugged mountainous island of Fernando Po, however different in aspect, have all exhibited the same remarakble uniformity in giving birth to the disease.

So long as the fever continued to make its appearance during the rainy season, excessive moisture was deemed one of the principal causes, but that theory has been abandoned since it has, on three or four occasions, appeared and raged with equal violence in the middle of the dry season. If we attempt to connect it with temperature, the range of the thermometer offers equally contradictory results, the disease having originated and prevailed nearly as often when that was at the minimum as when at the maximum. Variations in atmospheric pressure afford no clue whatever to the solution of the difficulty, for here, as in all tropical climates, the fluctuations of the barometer are exceedingly slight. No definite connection has ever been traced between the prevalence of any particular wind and the outbreak of the disease; the breeze blows over the same district in the healthy as in the unhealthy season. Besides, it seems entirely to negative the supposition that any of these can be more, perhaps, than mere accessories, when we find, from 1830 to 1836, the colony of Sierra Leone remarkably free from fever without any perceptible change in these respects." 27.

The composition of the atmosphere, during the prevalence of yellow fever in the command has not been closely examined. But much cannot be expected from any examination of the kind, for our readers know how signally it has failed elsewhere. The following observations are as painful as just.

" Though the primary cause of a disease which has created such mortality among the white troops on this coast may remain for ever involved in the same doubt and uncertainty as at present, a useful lesson may be learnt from the preceding details, as to the inexpediency of ever forming commuted-punishment men into corps for service in the Colonies. It is obvious that if such a corps is stationed in a healthy climate, banishment to it can scarcely be looked on as a punishment; but if sent to one exceedingly unhealthy, then the natural evils of climate are aggravated by despair, and that intemperance which despair too generally induces. In addition to the dread of sickness with which the soldier is impressed on his arrival, there is the certainty that, under no circumstances, will he ever be permitted to return to his native land, and the excesses to which this gave rise during the period when mortality was at its height in Western Africa are stated to have been such as to baffle description, and could only be expected from men absolutely weary of life, and driven by despair to the verge of madness. Setting all restraints at defiance, regardless of the warnings of their medical attendants, or the fate which a similar course of dissipation had accelerated in their comrades, every energy was directed to procuring the means of that intoxication which they vainly looked to as the best resource against care, and in search of which they fearlessly encountered the tropical rays by day, and the chilling dews by night. Punishment was of no avail; that of death itself was derided by men who knew that in such a climate their hours were already numbered; and to corporal punishment they had become so habituated that it lost its terrors, though it must have been inflicted with no sparing hand, when 12 deaths are recorded from it within a year.

Even had their crimes been such as to involve the utmost penalty of the law, banishment to such a climate was obviously far from a commutation of punishment; not a twentieth part of the criminals sentenced to death in the United Kingdom about that period were ever executed, the rest were sent to a climate in which their lives were likely to be prolonged to the utmost limit; but out of the same number of military culprits sent to the coast of Africa one-half generally died during the first quarter, and the average duration of life among the others did not exceed 15 months. Yet many of the crimes which led them to this coast were by no means of a heinous nature, either in a civil or military point of view, as it too often happened that those who wanted fortitude to bear a present punishment, though comparatively trifling, were glad to exchange it for one deferred, but of the nature of which they were ignorant." 27.

The reformation of the offender, was, of course, out of the question—the deterring from crime equally hopeless, for none returned to tell the tale to comrades, or to such as were likely to be culprits. To make matters worse, it was impossible to procure trust-worthy non-commissioned officers, so that those who should have checked, were often found promoting, insubordination. We cannot be surprised if, besides the frightful mortality that ensued among the unhappy wretches themselves, the lives and fortunes of the colonists were placed in jeopardy by their violence and their despair. Two conspiracies had well nigh proved successful. It is to be hoped, then, that the plan of keeping up a penal corps will be abandoned.

### II. On the Sickness and Mortality among Troops serving in the Island of St. Helena.

This "Atlantic Island" is situated between 15° and 16° of South Latitude, and 5° and 6° of West Longitude. It is about $10\frac{1}{2}$ miles in length, $6\frac{3}{4}$ in breadth, and has a superficial extent of above 30,000 acres. It is divided by a lofty chain of hills running in a curved direction from east to west, with several ridges branching off towards the north and south, and forming valleys of various extent, but all extremely contracted. At the entrance of one of these valleys, on the North West or Leeward side of the island, is James Town, the capital.

On each side of this valley the ground rises with great abruptness into two broken rugged eminences of considerable elevation: that on the west, termed Ladder Hill, is surmounted at the height of 600 feet by a battery, the ascent to which is so steep that it has sometimes to be made by a step ladder; the other is also of a similar precipitous character, consequently but little vegetation, except patches of furze and stunted shrubs, can find root, except towards the bottom of the valley.

The country becomes less wild and rugged, however, and the luxuriance of vegetation increases in proportion to the distance from the sea; most of the uplands are covered with verdure, many spots in the interior under rich cultivation, and at an elevation of nearly 1700 feet are two level tracts, called Longwood and Francis plains, the former of which is upwards of 1500 acres in extent. That part of the island is well-wooded, and affords good pasture, a few dwarf trees and shrubs are also scattered over the summits and sides of the high grounds in the interior, but, generally speaking, vegetation is scanty. Various small streams intersect the island, but there is no marshy or swampy ground.

The south-east trade wind affords a steady breeze, which, in these latitudes, is rarely disturbed by storms and gales, and brings with it a canopy of clouds sufficient to afford shelter from the vertical rays and to admit of labour and exercise being carried on with impunity, even during the heat of the day.

The temperature is found to vary very materially, according to the nature of the locality and different degrees of elevation. In James Valley, for instance, where the principal part of the troops are quartered, and where the reflection from the surrounding heights tends to augment the natural heat, the thermometer during the summer months sometimes rises as high as 85° and is generally about 80°, but at Plantation house, which enjoys an elevation of 1783 feet, the average at that season is much the same as in Great Britain. Owing, however, to the nature of the localities, a considerable variation of temperature may be experienced in a few minutes, the soldier, for instance, who ascends from James Town to Ladder Hill, exchanges the heat of the tropics for that of a temperate region. And in regard to moisture, the difference between the high and low grounds is still more remarkable. In James Valley the climate is very dry; in the upper regions of the interior it is remarkably the reverse; indeed, on the summits of the hills scarcely a day ever passes without rain. Thus, though it rained on 178 days of that year, at Plantation House, it only rained on 46 days at James Town, and the quantity which fell at the former station, was more than five times greater than at the latter. Electrical phenomena are said to be exceedingly rare.

It could scarcely have been anticipated that under the tropics, a situation could be found where the mortality among both the white and black population did not, on the average of a long series of years, exceed that of their respective native countries. There is no doubt, however, that this is the case in St. Helena. Major Tulloch presents an abstract of the deaths among the civil population, between October 1815 and September 1837, from which it appears that the annual mortality has averaged 1 in 48½, even including the class termed strangers, many of whom were seamen landed from vessels in the last stage of disease: in the United Kingdom it averages about 1 in 47½ of the population; consequently St. Helena must be healthier than Britain.

This is the more remarkable as a large proportion of the population are of the Negro race, who in general suffer to a great extent when transported from their native country; here, however, they are found to keep up, and even to add to their numbers, for though no importation has been permitted since 1792, they increased within the following 13 years, from 1512 to 1560, a feature which has never been observed in any other British colony.

There have been only two severe epidemics since the island's colonization. One occurred in 1718, another of measles, in 1807.

Prior to 1815, when this island was selected for the residence of the Ex-Emperor Napoleon, the garrison consisted of four companies of artillery, and a corps of infantry, raised expressly for the purpose, with two companies of invalids, all white troops, in the pay of the East India Company. A reinforcement of regular troops arrived with Napoleon, which was withdrawn shortly after his decease, and the island was again garrisoned solely by those of the East India Company till 1836, when, having been ceded to the crown, the colonial force was disbanded and replaced by troops of the line. During the period of the Company's occupancy, the force seems to have averaged about 800, exclusive of officers, while the deaths averaged from 15 to 16 annually, consequently the mortality must have been under 2 per cent., even including that of the invalid establishment, consisting of about 100 soldiers advanced in life; it is probable, therefore, that the mortality of the effective part of the force did not exceed the usual ratio in the United Kingdom.

After describing the barracks, Major Tulloch alludes to the bad rations which were issued from 1816 to 1822, fresh provisions being seldom served out, except to such soldiers as were in hospital. The daily diet consisted of a pound of salt beef or pork, and a pound of bread, with a pint of Cape wine, to which the soldier added such vegetables as he could afford from his pay, but, owing to the high price, these could never be procured in sufficient quantity to neutralize the constant use of salt-meat diet. The same rations were issued for some time after the arrival of the 91st, and it was not till nearly a third part of that corps had been in hospital and several deaths had occurred from diseases of the bowels, that measures were taken for improving them. Now there are allowed 2lbs. of fresh meat in the week.

From a table it would appear that among the King's troops, from the years 1816 to 1837, inclusive, the mortality averaged 35 per thousand annually.

The diseases from which the chief mortality has sprung have been, in order, Diseases of the Stomach and Bowels, Diseases of the Liver, Diseases of the Lungs, and Fevers.

*Diseases of the Stomach and Bowels.*—" More than a third," says Major Tulloch, " of the admissions and nearly two-thirds of all the deaths among the troops, have been from this class of diseases. Dysentery is the prevailing form, and is even more severe than in the West Indies; yet in vain do we look for any peculiarity either in the climate or the locality to account for it;—the heat, owing to a cloudy sky and constant breeze, is far from oppressive, the range of the thermometer is extremely limited, and except occasionally in passing from the narrow and confined valleys to the higher and more exposed parts of the island, sudden transitions of temperature are comparatively rare; the moisture, in the low grounds at least, where the troops are principally stationed, does not appear to have exceeded the usual average in similar latitudes, and there are neither marshes, forests, nor any excessive vegetation to which the most remote suspicion can attach of having operated unfavourably in this respect. Had these diseases been principally confined to the 66th Regiment, their prevalence might perhaps have been attributed to a large proportion of that corps having acquired a predisposition to them by previous service in the East Indies, but the 20th Regiment, though direct from Europe, lost precisely the same number, and had nearly twice as many cases in hospital; yet the East India Company's troops were almost entirely exempt, indeed, in some years, the mortality among them by all diseases together, was not higher than among the King's troops by diseases of the bowels alone.

It is remarkable, too, that the population of the island generally, appear to have enjoyed a similar exemption, for, as will be seen by reference to Abstract No. II. of Appendix, of 552 deaths, only 47, or a twelfth part, were from diseases of the bowels, while of 33,501 deaths among the inhabitants of Malta, 4920, or nearly a seventh part, arose from the same cause. Thus, so far as can be estimated from the years over which these observations respectively extend, the inhabitants of St. Helena suffer scarcely half as much from these diseases as the population of Malta, indeed, they do not appear to be more prevalent than in England, though as the classification in the Registrar-General's Returns differs from that which we have adopted, it is impossible to draw the comparison so clearly.

The connexion frequently found to subsist between hepatic derangement and affections of the bowels, might perhaps in some instances have given rise to the latter among the troops, but in that case, as indeed in every other in which their prevalence or fatal character is in any way attributable to climate, the same feature should have been manifested among the officers; whereas, of about 50

belonging to the two corps in garrison, from 1816 to 1822, the number under treatment for diseases of this class did not average above three annually, being scarcely a fifth of the proportion among the troops, and not a single officer died or was even seriously affected by them. The remarkable liability of the soldiers as compared with the officers, cannot here be attributed to the intemperance so common at other tropical stations, for it is stated by the medical officers, that no spirituous liquors were at that time allowed to be landed in the colony.

Neither can this liability be attributed in any material degree to the defective accommodation of the force, nor to the nature of the duties it had to perform during the residence of Napoleon, for in 1836, when the island was given up to the Crown, and when no such causes were in operation, these diseases began to manifest themselves shortly after the arrival of the troops, commencing with obstinate visceral obstructions which, unless removed by the use of medicine and a mild diet, ultimately terminated in severe dysentery. At this time also no such disease prevailed among the civil inhabitants, or the soldiers of the colonial corps which had been disbanded, nor were any cases of it observed among the officers.

In consequence of repeated representations to which the frequency of these diseases gave rise, a Board of Medical Officers was, in October 1836, directed to investigate the subject, who, after carefully examining into all the circumstances connected therewith, came to the conclusion that the health of the troops had manifestly been impaired by the constant use of salt rations; that in several, particularly those of a scrofulous diathesis, dysentery had been induced, and that when such persons were even fortunate enough to recover from a first attack, they generally experienced a recurrence of the symptoms immediately on returning to a salt-meat diet. Two days' fresh provisions per week were in consequence ordered for the troops, with the privilege of exchanging a portion of their salt meat for fish or vegetables. The beneficial effect of this alteration was shown by the cases of visceral obstructions being reduced to half their previous amount in the course of the following year, and now they are said to be comparatively rare."

It is only fair, then, to exonerate a climate which spared others as singularly as the soldier was attacked. And since, upon the one hand, the soldier was circumstanced differently from other classes in point of diet, and ceased to be affected in so marked a way by dysentery when that diet was changed, it is next to impossible to attribute to anything but diet the complaint he suffered under. This is confirmed still further, by what happened to the East India Company's regiments. They did not suffer, like the King's, from dysentery, a circumstance accounted for by most of them having formed connexions in the island, through whose aid they were in the habit of raising pigs, poultry, and vegetables, to improve their diet, which troops of the Crown, whose residence is always temporary and uncertain, had no similar opportunities of doing. The Company's troops appear also to have been in the habit of exchanging a large portion of their salt meat for fish and vegetables, and to have enjoyed the advantage of obtaining from the Government stores, tea, sugar flour, &c., considerably below the market price.

*Diseases of the Liver.*—Like that of Western Africa the climate of this island appears to exert an unfavourable influence on hepatic affections. They occur even more frequently and of a graver character than in the West Indies, though the temperature is lower and more uniform, and though other diseases are more rare.

Their influence in this island, compared with temperate latitudes, may be estimated from the fact, that of 552 deaths among the whole population, 16 were from diseases of the liver, being nearly . . . . . . 1 in 34½.

Whereas in England, the Registrar-General's Returns show that of 148,701 deaths, only 1,909 were from the same class of diseases, being . . . . . . . . . . . . . 1 in 78.

Supposing the composition of the population in regard to age, to be much the same in each, it may be inferred that diseases of the liver are more than twice as common in St. Helena as in England.

It may, perhaps, at first sight, appear singular, that the 1st Battalion of the 66th Regiment, which arrived at St. Helena from the East Indies, suffered less from hepatic disease than the 20th, which went direct from Europe. However, the former may be looked upon as seasoned.

*Diseases of the Lungs.*—As regards this class of diseases, St. Helena seems also remarkably healthy, the proportion of admissions and deaths among the military being not half so high as in the United Kingdom or Mediterranean stations, and the same feature is manifested in the fatal diseases of the population generally, as only 86 deaths occurred among them from diseases of the lungs in the course of five years, being in the ratio of $3\frac{2}{10}$ per thousand annually.

Whereas in Malta, the ratio of mortality by the same class of diseases among the population annually, was $5\frac{1}{8}$ per thousand

And in England, by the Registrar-General's Returns for the year ending December 1837, it was . . . . $5\frac{7}{10}$ ,, ,,

Similar deductions will be attained by calculating the proportion which diseases of the lungs bear to all other diseases among the population of these countries respectively, viz. of deaths in St. Helena, those from diseases of the lungs were 1 in 6½—in Malta, 1 in 5—and, in England, 1 in 4. It is from inflammation of the lungs that the exemption in St. Helena is most marked. But Major Tulloch remarks, that the last returns shew a much greater mortality from diseases of the lungs than occurred in previous years.

*Fevers.* "There can be no better proof that this class of diseases may be comparatively rare, even within the tropics, than that the admissions annually have been fewer, in the proportion of 71 to 75, than among an equal force in the United Kingdom.

This conclusion does not rest on the Returns of the troops alone, for in No. II. of Appendix will be found an Abstract of the fatal diseases among the whole population of the island, during a period of 6 years, which shows that the deaths by fever only averaged from 6 to 7 annually, in a population of 4500, being about $1\frac{4}{10}$ per thousand of all ages; whereas the proportion who died from the same class of diseases in England, during the year 1837, according to the Registrar-General's Returns, was $1\frac{3}{10}$ per thousand of all ages—the same diseases thus producing nearly similar effects in each."

St. Helena has been hitherto exempt from epidemic fever. Yet, in 1823, it prevailed alarmingly in the neighbouring island of Ascension, still more rocky and more free from wood and marsh than St. Helena.

Of deaths in St. Helena, 1 in 14 were from fever—in Malta, they were 1 in 12—and in England 1 in 16.

From a table it appears, that, though the number attacked by sickness was shown to have been fewer, in the proportion of 738 to 929, the diseases were of a much more lingering nature than in this country, owing no doubt, to the large proportion of visceral affections, which are described as being exceedingly intractable and difficult of cure.

Another table shows that the months of August and September, though the most sickly in the Mediterranean and America, are here the reverse, and that March and April, the healthiest in these Colonies, are here the least so. As St. Helena is to the southward of the Line, this is readily explained by the seasons being reversed, so that any atmospheric agency causing an increase of sickness is likely to come into operation at a directly opposite period to what has been observed at stations in the Northern Hemisphere.

On the whole it must be admitted that St. Helena is remarkably healthy. When we compare it with the Coast of Africa that we have left, we find the great points of difference to be a more moderate temperature, and an absence of that long-continued wet season, which, proximately or remotely, can hardly fail to be a source of disease. The want of much vegetation and of marsh may be paralleled indeed in some of the African stations of the deadliest character. But those stations, if not wooded or wet, are, to say the least, not so remote from a continent which is both, and is eminently rife in the seeds of fever, as the sea-girt island of St. Helena. In the latter then, we have, not one cause only of salubrity, but a combination of many—a dry soil—an open surface—a moderate heat, a comparatively temperate climate—and the regular breeze of a trade wind. If the points of difference between St. Helena and the African Coast or those islets which may be almost deemed a part of it, are so considerable, we need not be astonished at an equally considerable difference of salubrity.

### III. ON THE SICKNESS AND MORTALITY AMONG THE TROOPS AT THE CAPE OF GOOD HOPE.

In this Command there are two Military Districts, one comprising Cape Town and its vicinity, the other the stations on the eastern frontier of the colony. As the latter is at the distance of nearly 500 miles from the former, and exhibits considerable difference in physical aspect, climate, and salubrity, Major Tulloch gives, first, a general description of the colony, and then investigates separately, the statistical details of each.

The south-western extremity of Africa, generally termed the Cape of Good Hope, lies in lat. 34° 22′ S., long. 18° 24′ E., and consists of a detached mass of high rocky mountains, connected with the mainland by a flat sandy isthmus, forming a peninsula about 36 miles long and 8 broad, bounded by Table Bay on its western and False Bay on its eastern extremity.

The colony of the Cape of Good Hope, however, is of much vaster extent, reaching about 560 miles in an easterly direction from Cape Point, as far as the Keiskamma River,* and about 220 in a northerly direction to the Ga-

* By the recent alterations in the boundaries, the colony now extends only to the Great Fish River.

riep or Orange River, including altogether a surface of above 125,000 square miles.

The whole colony is intersected by three chains of mountains, running like steep walls from east to west, and enclosing belts of land of varied character. That nearest to Cape Town, between the coast and the most southerly chain, is tolerably fertile and watered by numerous streams, is in many places well wooded, and on account of its proximity to the sea enjoys a more mild and equable temperature than the interior. Between the most southern and second range called the Zwarteberg or Black Mountains, the country presents a great extent of arid plain, occasionally interspersed with small farms and plantations, wherever a sufficiency of water can be found to render the soil available for cultivation ; while between the second and third range extends an arid, barren, and almost uninhabited desert, 300 miles in length, and 80 in breadth, known by the name of the Great Karroo.

These mountain ranges, as well as the general surface of the country, rise gradually towards the interior, the plain of the Great Karroo being nearly 1,200 feet above the level of the sea, and the range of mountains which bounds it on the north, termed the Nieuwveld, attains in some places the height of nearly 10,000 feet.

There is likewise a gradual ascent, by successive hills and terraces, from the western coast towards the last-mentioned range, and this portion of the country exhibits the same wild desert character as the great Karroo, except in a few spots at the base of the mountains, or by the banks of springs or rivulets, where the supply of moisture adapts the soil for pastoral and agricultural purposes.

Between the extremity of the third range of mountains, and the vicinity of the Orange River, which forms the northern boundary of the colony, a rocky desert of immense extent intervenes, equally destitute of soil and water, and occupied only by the wandering bushmen, and a few tribes of Hottentots.

At the eastern extremity of the Great Karroo, the mountains gradually decline towards the sea, and a fine extent of pastoral country, intersected by several streams and rivulets, opens to the view. This forms what is termed the Eastern Provinces, which, prior to the late alteration of the boundaries, extended as far as Keiskamma River.

The cause of so large a portion, amounting probably to nine-tenths of this extensive colony, being totally unproductive, and available for no useful purpose, arises, not so much from any deficiency in the soil, as from the want of water. In many of the desolate regions bordering on the Great Karroo, three years have sometimes elapsed without a drop of rain, and even in the more favoured districts of Albany and Uitenhage, the supply is exceedingly limited and irregular.

When rain does fall it is in torrents, accompanied with thunder-storms, filling the river-courses, overflowing their banks, and soon leaving the country as arid as before. Nature has protected the colony by the desert to the north. But on the east the Caffres, have rendered an extensive line of military posts requisite.

A. *The Cape District.*

The country to the east of Cape Town is an extensive, and for the most part, barren sandy plain, terminated at the distance of 30 or 40 miles, by the rugged and abrupt mountains of Hottentot Holland. At the extremity of this plain lies the district of Stellenbosch, which, possessing abundance of water and a good soil, presents a pleasing exception to the general sterility. The country to the distance of 10 or 12 miles south from Cape Town is also highly cultivated, well wooded, and thickly inhabited.

The town is situated on a gravelly plain at the west side of Table Bay, having a gentle ascent towards the foot of three barren precipitous mountains which, stretching from the north-west to the north-east, form an amphitheatre, having its front to the bay.

As the surface of these mountains is composed principally of sand-stone, which reflects the solar rays during the day, and gives out a portion of its acquired heat during the night, the town is, from its position, subject to a much higher temperature in summer than is usual in similar southern latitudes. This will be apparent from the fact, that a thermometer which, a little after mid-day, stood at 86° in the shade, rose to 136° on being hung against a wall exposed to the sun and breeze. When unsheltered, it occasionally ranges, during the middle of summer, from 105° to 110°.

The want of rain and moisture, which renders the greater part of the interior a barren desert, is but little experienced in the Cape district. The average number of rainy days, during a series of years, was 75, and the quantity which fell averaged 41 inches annually, but we possess no accurate measurement of the relative quantity in each month.

The prevailing winds at Cape Town are from the south-east and north-west; the former is most common during summer, and, blowing over the sandy flats between the town and Simon's Bay, is usually sultry ; the latter, being a sea-breeze, is cold, chilly, and often accompanied by heavy falls of rain, and violent gales.

South-westerly winds prevail during spring and autumn, and coming across the wide expanse of the Southern Ocean, are generally surcharged with moisture, which wraps the summits of the mountains over Cape Town in dense fogs. As the upper stratum of the air becomes cooled, these rapidly descend in tempestuous blasts, causing an immediate reduction of temperature, with an equally suddden transition from an extremely dry to a damp raw atmosphere.

A detachment is furnished from Cape Town to a small rocky island in the entrance to Table Bay, called Robben Island. There is another military station at Simon's Town, about twenty-two miles distant, situated at the foot of a steep mountain, on the shores of a bay in which the shipping generally take refuge during the winter months, when the wind blows with such violence into Table Bay as to render anchorage there exceedingly dangerous. The climate of this station is neither so variable nor so liable to sudden transitions as that of Cape Town, and the thermometer is lower by several degrees. The town is well sheltered from the violence of the north-west winds, and those from the south-east coming direct from the ocean, are cool and pleasant. As it rarely blows from any other quarter, and as both these winds are laden with moisture, considerably more rain falls than at Cape Town.

Neither the variable climate of the Cape District, nor the high range of temperature during the summer, seem by any means prejudicial to health, for in 1833, the deaths were only 681 out of a population of 31,167, being 1 in 46, while in the United Kingdom, according to the last census, the proportion was 1 in $47\frac{1}{2}$. When it is taken into view that among the former are included the deaths of many invalids who arrived at Cape Town in the last stage of disease, there can be little doubt that, so far as regards the resident population, the climate is at least as favourable to the constitution as that of Britain.—It may be stated as a further proof, that in the neighbouring districts of Swellendam, Stellenbosch, and Worcester, where the deaths were not so liable to be increased by the arrival of invalids, the mortality for 1833, was only 707, out of a population of 47,071, or 1 in 67, being a lower ratio than in the healthiest counties in this kingdom.

The duties of the troops are, on the whole, light—the barracks respectable, save at Cape Town—where they are too hot in the summer—the rations not bad, save that there is no supper, and 19 hours are passed without food, a practice reprehensible in a country where ardent spirits can be so readily procured.

*Mortality.*—With casualties, this amounts to $15\frac{1}{2}$ per thousand, being very nearly the same as among the Dragoon Guards and Dragoons in the United Kingdom, which, combined with the facts already adduced in regard to the civil population of the Cape District, affords sufficient evidence of the general salubrity of that part of the Colony.

It will be observed that both the sickness and mortality have been very uniform in every year; the highest ratio was in 1825, when fever and dysentery were very prevalent. This uniformity in the results of each year, is, in a great measure, owing to the absence of epidemics. For cholera has not hitherto found its way there, and influenza has been milder than in any quarter of the globe. Intemperance, however, is very common, and though the young soldier escapes, among the older ones the ratio of mortality increases with great rapidity.

From a table it appears that, out of 22,506 admissions, 14,661, or very nearly two-thirds of the whole, are of that description which seldom prove fatal, and for which, indeed, if the soldier had been left to his own option, it is by no means probable he would have submitted to the confinement of hospital. The same feature, and to nearly the same extent, was noticed in recording the diseases among the Dragoon Guards and Dragoons in the United Kingdom. This always constitutes a marked distinction between the diseases of tropical and temperate climates, and shows that the number of admissions into hospital among the troops is of little use as an element for estimating the salubrity of a station, or the probable efficiency of a garrison, unless accompanied by an accurate specification of the diseases by which they have been caused.

The severe diseases prevail in the following order—diseases of the stomach and bowels—diseases of the lungs—fevers—rheumatic affections.

*Fevers.*—Comparing these with the average extent of sickness and mortality from the same cause among troops in the United Kingdom, we find them nearly to correspond, the admissions being as 88 to 75, the deaths as

1·9 to 1·6. The extreme rarity of fevers of the intermittent and remittent type, is particularly striking; indeed, among the inhabitants they are said to be altogether unknown.

"The sandy nature of the soil, the rocky formation of the under strata, the total absence of marsh, and the comparative scarcity of wood and forest in Cape Town and its vicinity, have all been assigned as causes of this marked exemption from remittent and intermittent fevers; but we shall hereafter have occasion to show that in other colonies, the Mauritius for instance, these diseases are equally rare, at stations of which the physical character is directly the reverse."

Most of the cases of common continued fever are said to have arisen from the immoderate use of Cape brandy. At all events this did not add materially to the mortality. Except in 1825, the number of fever cases has been remarkably uniform. On that occasion the disease was principally confined to the 55th and 98th regiments; of the former, 73 were attacked out of a strength of 366; of the latter, 113 out of 556, being exactly a fifth in each instance. The 49th, though also at Cape Town, did not suffer more than usual, nor did the disease extend to the troops at Simon's Town. It is said to have been somewhat different from the usual form prevalent in the garrison, assuming in most cases a mild typhoid character, and was supposed to have originated with the 98th regiment, a corps recently raised, and which had then newly arrived in the garrison.

Eruptive fevers have been exceedingly rare.

*Diseases of the Lungs.*—In most of the Medical Reports, the prevalence and fatal character of this class of diseases is strongly commented on, under the impression that, owing to the sudden changes of temperature and violent gusts of wind to which Cape Town is exposed, the troops are more subject to them than in other colonies. We find, however, the reverse of this to be the case. For, on comparing this and other Reports, it turns out that, the deaths annually per 1000 of the strength from all diseases of the lungs in the Colonial Stations were the following:—Windward and Leeward Command, $10\frac{4}{10}$;—Jamaica, $7\frac{5}{10}$;—Gibraltar, $5\frac{3}{10}$;—Malta, 6;—Ionian Islands, $4\frac{8}{10}$;—Bermuda, $8\frac{7}{10}$;—Canada, $6\frac{7}{10}$;—Nova Scotia and New Brunswick, $7\frac{1}{10}$; and Cape District, $3\frac{9}{10}$.

"This shews better than any other description of evidence can possibly do, how erroneous is the impression that the climate of this district has any peculiar tendency to excite or aid in the development of pulmonary affections. On the contrary, the aggregate mortality from them is less than in any of the colonies above referred to, and the degree of prevalence is greatly under the average, though, according to the generally received opinions on such subjects, the climate might be supposed much more likely to induce them.

This error has probably originated in there being hitherto no document whereby a medical officer could compare the influence of the same diseases in other colonies with that in which he is serving; consequently, in healthy climates, where the admissions and deaths by diseases of the lungs must always form a considerable proportion of the aggregate sickness and mortality, their influence is apt to be over-rated, while in unhealthy climates, where they form a comparatively small item in the general mass, the reverse is the case, though they may in reality be more prevalent and fatal."

Facts, like this, are invaluable to medicine, and we must repeat that these

Reports go very far towards shaking some of the notions which have taken deep root in the minds of medical men.

Complaints of the lungs have preserved a very great uniformity in the several years through which the Report extends.

*Diseases of the Liver.*—Though this Colony enjoys a happy exemption from other tropical diseases, those of the liver are rather frequent in occurrence; the proportion of admissions and deaths is nearly the same as in Malta or the West Indies, 22 per thousand being attacked, and $1\frac{1}{10}$ per thousand of the strength dying annually by them. Neither here, nor on the eastern frontiers of the Cape, however, do they produce the same fatal effects as at St. Helena or the West Coast of Africa, though the temperature, particularly on the frontiers, ranges higher during summer than in either of these Colonies.

*Diseases of the Stomach and Bowels.*—This class of diseases does not in the aggregate exhibit any great degree of prevalence, the ratio of admissions compared with what has been observed among troops in the United Kingdom, being relatively as 126 to 94. It is even considerably lower than in British America; but with this marked distinction, that there, affections of the bowels show themselves chiefly in slight attacks of diarrhœa which yield readily to remedial measures, whereas in this colony, nearly one-half of the cases assume the form of dysentery, which, after repeated relapses, become chronic, and in that stage are so apt to prove fatal that the deaths average 1 in $4\frac{1}{2}$ of the admissions.

This distinction will account for the mortality from diseases of the bowels being thrice as high as in the North American stations, and nearly five times as much so as in the United Kingdom. In that respect the troops at the Cape seem nearly on a par with those in the Mediterranean. Much of their sufferings from these diseases have been attributed to habitual intemperance, the want of due precaution when labouring under slight attacks, and an unguarded indulgence in the use of fruit, which the colony produces in great abundance.

It has been stated that regiments newly arrived suffer, in this respect, to a greater extent than others, but, though that was the case in 1822 and 1825, it does not appear to have been so, on prior or subsequent occasions, when changes took place in the garrison. Very few cases, however, particularly of dysentery, have occurred from 1831 to 1836, a period during which no new corps arrived, and this seems to favour the idea of the tendency to these diseases being diminished, as the troops acquire experience in guarding against the causes likely to induce them.

*Diseases of the Brain.*—"This class of diseases exhibits nearly the same degree of prevalence and severity as in the North American colonies. A large proportion of the cases are said to have been, directly or indirectly, attributable to intemperance, but here this vice does not seem to produce the same baneful effects, by giving rise to delirium tremens, as among the troops in North America, where that disease is nearly tenfold as common. If the relative prevalence of delirium tremens throughout all the colonies is investigated, it will be found rare wherever wine is procurable at a moderate rate, compared with stations at which spirits form the principal intoxicating medium; a circumstance which should lead to the sale of the latter being placed under more rigid regulations than the former, as having greater tendency to induce permanent injury of constitution."

In noticing the various minor complaints, Major Tulloch dwells on the tendency of the climate of the Cape to induce rheumatic affections. In this respect, it is worse than the United Kingdom, or any of the colonies which have yet come under our observation. These diseases are also said to be still more common among the civil inhabitants, and are attributed to the winds, during spring and autumn, being so much surcharged with moisture, and blowing, in such violent gusts, from the mountains. The latter of these causes may, perhaps, have some effect, but the influence of the former appears very doubtful, because, on the eastern frontiers, which suffer under an extreme want of moisture, rheumatic affections are found to be nearly as common as at Cape Town.

Venereal diseases are rather more numerous than in the United Kingdom, and some of the cases are exceedingly obstinate of cure, especially when contracted from Hottentot females, whose dirty and dissolute habits are said materially to aggravate their virulence. No sanatory regulations can be said to be established.

In the winter and spring of 1825, erysipelas prevailed to a great extent in the 98th regiment, but did not attack any of the other corps; there were in all 22 cases, 3 of which proved fatal. No satisfactory reason could be assigned for its appearance, nor did it seem at all connected with any atmospheric influence observable at that period. The disease ceased as summer approached, nor has it since been common at the station.

### b. *Eastern Frontier District.*

The troops employed on the Eastern Frontier of this Colony are principally stationed in the Province of Albany, which is bounded on the west by part of the province of Uitenhage; on the north by a lofty, and in many parts inaccessible range of rocky mountains, rising to the height of from 7000 to 10,000 feet; on the south by the sea; and on the east, by the Great Fish River, and territory of the Caffres.

In the province of Albany, the mountains break into a pleasing succession of hill and dale, forming a rich pastoral district. The soil in most parts is alluvial, but on approaching the sea, where the surface declines into a succession of level plains, it becomes light and sandy.

The aspect of this part of the colony is described as being extremely pleasing; the high grounds are in general thickly covered with bush, but the low grounds are open, and occasionally dotted with clumps of mimosa, which give them the appearance of an extensive park. Though intersected with streams and rivulets, these frontier provinces frequently labour under a great want of water. Except in the vicinity of the sea, but little dew is deposited at night; and as rain only falls in any quantity during the months of November, December, and January, the herbage is frequently destroyed for want of nourishment. Even the water requisite for the use of the troops is sometimes difficult to be procured; and, in consequence, the positions occupied by them along the frontiers have been selected, not so much with reference to the facility the ground may happen to afford for the purpose of military defence, as from its vicinity to some of the rivers ensuring at all times an adequate supply of this necessary of life.

The climate in different parts of the frontier varies very materially ; about Graham's Town and near the sea coast the winter nights are sharp and clear, accompanied by slight frosts, while the summer heat, though sometimes intense, is generally tempered by a cooling breeze. At some of the posts, however, which do not possess this advantage, and where the wind is heated by the arid and sandy surface of the interior, the temperature during summer is excessive. On the Keiskamma and Great Fish Rivers, for instance, the thermometer about noon has been frequently known to range for several weeks, from 105° to 110° in the shade, and from 135° to 140° in the sun ; and even during several months it has seldom been under 95° at that hour.

This portion of the colony, however, is, throughout the whole year, subject to very sudden transitions of temperature ; the thermometer in summer has been known to fall from 110° to 64° in the course of a few hours, and, in winter, though it is often as low as the freezing point at night, it sometimes rises to 70° or 80° at mid-day. The degree of heat in summer is in a great measure regulated by the quantity of rain in the preceding season ; if the fall has been plentiful, the summer is comparatively cool, if scanty, the reverse.

Notwithstanding the extremely high temperature of this climate, its salubrity is probably unequalled in any portion of the globe. As a proof, Major Tulloch states, that, in the three adjoining districts of Somerset, Albany, and Uitenhage, the deaths in 1833 did not amount to more than 327, in a population of 30,000 being only 1 in 91, which is much lower than has ever been observed even in the very healthiest districts of Great Britain. It is no doubt, possible, that from the effect of immigration, there may be a greater proportion of persons in the prime of life in these provinces, than in a country of which the population is stationary ; but even making all due allowance for that contingency, we have sufficient evidence that the climate of these provinces is, in an eminent degree, favourable to the European constitution.

The duty of the regular troops, even in years when no warfare prevailed on the frontier, has generally been of a more varied and active description than in other colonies. As occasion required, they have been employed in erecting forts, building barracks, making and improving roads, escorting stores and supplies from the sea-coast to the different stations in the interior, guarding cattle in the field to protect them from the inroads of the Caffres, or patrolling the country in various directions in search of them when stolen. Though these duties have necessarily caused much exposure under an excessively high temperature, they do not appear to have militated against the health of the troops.

As, in 1834, there was an irruption of Caffres, and the posts were altered while casualties from warfare necessarily ensued, Major Tulloch stops at that year, and the Report refers to stations and seasons antecedent to that date.

*Port Elizabeth or Algoa Bay*, being the principal sea-port of the frontier districts, first claims our attention. It is nearly 500 miles from Cape Town ; and as this distance, if travelled overland, would prove exceedingly harassing and injurious to troops, from the arid nature of the country through which they would have to pass, all detachments and reliefs for the force on the frontier, are sent by sea from Cape Town to this port, whence they are marched into the interior. Military supplies are forwarded by the same route; consequently the station always requires the presence of a small garrison, to aid in unloading the Government stores, and protecting them from depredation. On this duty an officer and 30 men have usually been employed, under the medical superintendence of an assistant-surgeon. The barrack accommodation is so-so. A good hospital is to be built, for the sake of patients sent from the interior, who often derive much benefit from the change of air. From its proximity to the sea, the temperature during summer is nearly ten degrees lower than in the interior. The soil enjoys a greater share of moisture, dew falls more abundantly, and the climate has always had a high character for salubrity.

About 100 miles in a north-easterly direction from this port, lies—

*Graham's Town*—The capital of Albany, and head-quarters of the eastern division of the colony. Though this town is situated at the foot of a range of hills which intersects the province from north-west to south-east, yet, as there is a gradual ascent all the way from Port Elizabeth, it stands at least one thousand feet above the level of the sea. One of the chief branches of the Cowie river flows through it, and the soil in the vicinity is good, but there is little cultivation, owing to the supply of rain being very precarious; except in seasons of extreme drought, however, this part of the colony produces excellent pasturage.

The force quartered here has varied according to circumstances, but has generally amounted to about 400 men, principally infantry of the line, and a few artillery.

This station is subject to a high degree of temperature in summer, and to comparatively severe cold in winter. Snow is rare, but ice is often formed of considerable thickness.

The influence of the hot winds which blow over the sandy surface of the interior, is occasionally experienced here during summer, when the air becomes so arid, as to create a parched disagreeable sensation in the mouth and organs of respiration, accompanied by restlessness and slight febrile excitement. Dew is very rarely deposited, and there is a deficiency in the supply of rain, though not so great as at some of the stations further to the eastward. Notwithstanding these peculiarities, however, the climate is uncommonly salubrious, and severe or protracted indisposition is of rare occurrence among the troops.

*Fort Brown*—Nearly 18 miles north-east of Graham's Town, is situated on a rising ground close to the banks of the Great Fish River, and surrounded on every side by bare rocky hills, of slight elevation. The soil along the banks is of good quality, affording excellent pasturage, but beyond that, is light and sandy, and the surface either barren or thickly covered with bush. The station suffers much from want of rain; indeed, during a great part of the year the bed of the river is nearly dry. Owing to this circumstance, the supply of vegetables is very scanty, and, having to be brought from a considerable distance, they are too dear to admit of being in general use among the troops.

The strength of the garrison has varied from a captain's to a subaltern's party.

Owing to this post being surrounded by bare rocky ground, and in the vicinity of an arid and sandy expanse of country, the temperature is very high during summer; the winds from the interior are often insufferably hot, and succeeded by close sultry evenings equally oppressive. During the winter the breezes are cool and refreshing, and if there has previously been a good supply of rain, that season of the year is described as very pleasant.

On *Caffre Drift*, the *Gualana Post*, and *Fort Wiltshire*, it does not seem necessary for us to dwell, all being now abandoned. We pass on to—

*Fort Beaufort.*—This fort stands on a small elevation about 50 feet above the Kat, one of the principal tributaries of the Great Fish River, having some gently rising ground in front, and backed by a high range of mountains in the distance.

The Kat seldom dries up even during the hottest season, nor is it liable to overflow like several of the rivers we have described. The banks, as well as the numerous ravines with which each side of the river is indented, are clothed with wood, but in other parts, the country is generally open; the soil is composed of a rich mould, and as it enjoys the benefit of fertilizing showers during 60 or 70 days in the year, vegetation is more abundant than at the other stations; there is little cultivation, however, of any kind in the vicinity.

The climate is much more temperate than at Fort Wiltshire; the hot winds are not so oppressive, though the summers are occasionally sultry. During the depth of winter, frosts are frequent, but never severe, and the station enjoys a high character for salubrity.

About 130 men *were* stationed there. But the force *will* be much larger; and suitable barracks, &c. are to be erected.

*Fort Armstrong.*—This fort stands near the banks of the Kat River, on a high promontory, which, by the windings of that stream and its tributaries, is converted into a kind of peninsula, accessible on one side only. From the facility of defence thus afforded, and the circumstance of nearly 4000 Hottentots having settled in the vicinity, the position has of late grown into considerable importance.

This part of the country being a natural basin, enclosed on every side by mountains several thousand feet in height, has the advantage of being well watered by numerous rivulets from the high ground which are never dry even in summer. These rivulets being also very tortuous in their course, afford ample scope for irrigation, by means of which cultivation is carried on to a considerable extent.

Being surrounded by bare precipices, and sandy hills, the temperature of the fort is very high in summer. Hot winds from the north-west are occasionally experienced at that period, but in winter the cold is often severe, especially during the night, and so sudden are the changes of temperature, that the thermometer has been known to range from the freezing point to 80° in the course of a few hours. The climate differs from that of the stations to the southward, being more damp, and subject to heavy rains and thick fogs. The bottoms of the valleys are frequently moist and in some places marshy, giving rise to exhalations, but in no respect injurious to the health of the troops.

The *rations* issued to the troops on the frontiers are the same as at Cape Town, but, though cattle and sheep are plenty, the meat is sometimes so lean, at stations subject to long drought, as to render the issue of an additional quantity necessary. Except at Port Elizabeth and Graham's Town, much inconvenience has also been experienced from want of vegetables; and, where the supply is dependent on the wheat grown in the vicinity, the bread is frequently of very indifferent quality. Sometimes, when vegetables are very scarce, a quantity of rice is issued to the soldier as part of his ration.

Major Tulloch gives a table, shewing the admissions into hospital and deaths among the white troops serving on the frontiers of the Cape, from which, as well as from the mortality among the civil population, there can be no doubt that this portion of the colony is more favourable to health than the United Kingdom—the ratio of admissions into hospital, annually, being relatively as 866 to 929, and the deaths as $9\frac{8}{10}$ to 14. Deducting deaths from accidental violence, we find the aggregate mortality from all causes, 12 per thousand annually, which is the lowest yet observed in any colony.

On comparing the deaths from various diseases in the Colony with those from the same diseases in the United Kingdom, it is evident that the low ratio of sickness and mortality among the troops in this part of the colony has been mainly attributable to the extreme rarity of diseases of the lungs. Pneumonia and consumption, in particular, are still less common than at Cape Town, though, from generally received opinions as to the influence of sudden atmospherical vicissitudes in inducing them, the reverse might have been anticipated, seeing that at some of the stations the thermometer in Summer has been known to range from 110° to 64°, and in Winter from 75° to 32°, in the course of a few hours. This exemption cannot altogether be accounted for by the absence of moisture and extreme aridity of the soil, because the same diseases have been found twice as prevalent and twice as fatal in Malta, where, during the Summer months at least, similar causes are in operation, with the supposed advantage also of a high temperature little subject to extreme variations.

Fevers are still more rare and less productive of mortality than at Cape Town: in fact, so far as we have yet been able to ascertain, no part of the world seems to enjoy so great an exemption, particularly as regards those of the remittent and intermittent types. Though this exemption might in some measure be anticipated, from the absence of all marshy ground, and the otherwise favourable nature of most of the localities in which the troops are posted, yet that character will not apply to all. Fort Wiltshire, Caffre Drift, and Fort Brown, for instance, are situated close to the bank of a river, which being either dry or stagnant during summer, might be expected, under a high temperature, to give rise to exhalations such as are supposed to induce febrile diseases in the vicinity of *fumiares*, or beds of mountain torrents, in Spain, Portugal, and the Ionian Islands.

Diseases of the brain prove only half as fatal as at Cape Town, and are even less so than in the United Kingdom; their rarity is the more remarkable considering the exposure to which the peculiar nature of their duties has often subjected the troops, under a temperature which, during summer, generally ranges from 95° to 110° in the shade at midday, and when the soldier has frequently no better protection from its influence than the rude wicker huts constructed by himself at the out-stations.

Though the foggy damp atmosphere, and sudden gusts of wind, to which the prevalence of rheumatic affections in the Cape District has been attributed, are but little experienced on the frontiers, that class of diseases is exceedingly common there, indeed twice as much so as in the severe climates of Nova Scotia and New Brunswick; nor are they confined to the white troops alone, for even the Hottentots suffer to a still greater extent.

In 1835 and 1836, a disease of rare occurrence in the Colony prevailed. It was towards the end of June, 1836, that very decided symptoms of scurvy began to manifest themselves among part of the 75th regiment at Fort Armstrong, and subsequently extended to most of the other stations along the frontier: the total number of cases reported, either as scorbutus or purpura, were 134, of which 4 proved fatal; the others readily yielded to change of air, with improved diet and accommodation.

Considerable difference of opinion exists as to the causes to which this disease was attributable, but the circumstances in which the troops were then placed seem sufficient to account for it. Their active service had recently terminated—a period when troops are generally most amenable to disease. The weather was exceedingly variable, the thermometer ranging from 80° at midday to 45° at night, and though the latter is deemed very cold in this climate, the huts were without fire-places, the floors were of clay or earth, and the soldiers generally slept on them without stretchers, and with only one blanket. Except a few pumpkins occasionally purchased from some of the neighbouring tribes, no vegetables could be procured, and the ration of rice, issued in lieu of them, was found a very inadequate substitute. The meat too, had, for nearly twelve months previous, been of very inferior quality, and the water was bad, as no rain had fallen for about five months. The officers and non-commissioned officers, who had the means of obtaining additional comforts, were exempt from the disease, and it did not all affect the Hottentot troops.

* *Hottentot Troops.*—Major Tulloch very justly remarks that, " by extending these statistical investigations over the numerous colonies of the British Crown, not only are we enabled to ascertain the influence of their varied climates on the constitution of our own countrymen, but, in many instances where native corps have been formed, we can trace the diseases, and estimate the mortality, to which the aboriginal inhabitants are subject, and thereby supply a most important desideratum in the physical history of mankind, which could probably never have been obtained, with equal accuracy, from any other source.

We have in this way ascertained the diseases and mortality of the negro, both on his native continent and the foreign soil of the West Indies; we now proceed to a similar inquiry regarding the Hottentots, composing the Cape corps, who, as already stated, are quartered along the eastern frontier of this colony."

Till 1828, this corps consisted of from 350 to 500 cavalry and infantry, but, in that year, the infantry was reduced, and the cavalry, being the most effective species of force for service on the frontier, was augmented to about 250. Conscription was at first resorted to, but the force is now kept up by voluntary enlistment. The Hottentot soldiers are generally of low stature, seldom exceeding 5 feet 4 inches, but, though of very slight conformation, with narrow chests, they have shown themselves capable of undergoing great bodily fatigue. The disinclination at first manifested to the duties of military life gradually wore off, under the influence of a mild discipline, and the great majority of them have since become quiet steady soldiers, very submissive to authority, and of the utmost service for the duties of the frontier. Being possessed, in an eminent degree, of that acuteness of vision and quick perception, for which aboriginal tribes are sometimes so remarkable, they can, by indications totally imperceptible to an European eye, discover and follow up the traces of cattle stolen by the Caffres, which they frequently succeed in recovering, when the exertions of other troops would, in all probability, have proved unavailing.

If any of the colonists are reported to have lost cattle, a party of this corps is ordered out to recover them, and, if not thus employed, they are generally engaged in patrolling that portion of the frontiers which extends between the out-stations, and is most open to the incursions of the Caffres. They have thus little intermission of duty during the day, and every third night is passed on guard or picquet. This duty, though severe, is well suited to men of a wandering disposition, and who, probably, would never submit to the confinement and restraint of a garrison life. They are mounted on small hardy horses, well adapted for the nature of the service in which they are engaged, and the frequent privations of water and fodder they have to endure.

Intemperance in the use of spirituous liquors is, unfortunately, the besetting sin of the Hottentot, as well as the British soldier, and to this the former adds a pernicious mode of exciting intoxication by smoking hemp in large quantities. He is also apt to indulge in great excesses of animal food, when it can readily be procured. His pay and rations are the same as those of the British soldier, but occasionally the allowance of meat has been increased to two pounds a day, when there was a difficulty of obtaining a sufficient quantity of vegetable nutriment, or when, from the scarcity of fodder, the meat was of very inferior quality.

Notwithstanding their frequent exposure, and the harassing nature of their duties, the sickness and mortality among this description of troops has been exceedingly low, not exceeding $12\frac{1}{2}$ per thousand. Febrile diseases are still more rare among the Hottentots than the White Troops; indeed, it may be doubted whether any race of men in any quarter of the globe would be found to exhibit so great an exemption from them. All the cases under treatment, except two, were of the common continued type, and so slight that only one death occurred in 270 attacks.

The ratio of admissions and deaths by diseases of the lungs appears to be higher than among the white troops. This has been attributed to the narrowness of chest, already stated as a peculiarity in the conformation of the Hottentots, and also to the seeds of disease being frequently sown in early life when, in their capacity of herdsmen to the colonists, they are exposed, with a very scanty supply of clothing, to the inclemency of the weather. These causes, however, cannot have operated to any great extent, as the prevalence of inflammation of the lungs among them, compared with the white troops, has been only as 24 to 15; of catarrhs, as 75 to 60; while the proportion of each race attacked by consumption has been to within a fraction the same, viz. $3\frac{1}{3}$ per thousand annually. The proportion of deaths by that disease is certainly higher among the Hottentots; but it must be kept in view that many of the white troops may have been invalided, whose deaths at Cape Town or on their passage home cannot be traced; whereas the Hottentots remain under treatment till the disease terminates in death or recovery.

Notwithstanding their frequent intoxication and constant exposure to a temperature which, in summer, is rarely equalled in any part of the world, these troops enjoy a very great exemption from diseases of the liver; the same remark applies also to diseases of the brain, of which so far as can be traced, no fatal case has occurred during the whole period under observation.

The diseases from which they suffer most are those of the bowels, whereby nearly half the deaths in hospital have been occasioned. All the fatal cases, but one, have been from inflammation or dysentery, which though only in a slight degree more prevalent among this class of troops than Europeans, are decidedly of a more serious character, being frequently of long standing before they come under treatment, owing to the disinclination of the Hottentots to subject themselves to the confinement and restraint of hospital.

The most unhealthy quarter of the year at the Cape is from January to March.

### IV. On the Sickness and Mortality among the Troops serving in the Mauritius.

This island is of an irregular oval shape, 36 miles in length, and from 18 to 27 in breadth, with a superficial extent of nearly half a million of acres. It is situated in the Indian Ocean, about 500 miles to the eastward of Madagascar, from 70 to 80 north-east of the island of Bourbon, and lies in Lat. 20° 9′ S., Long. 57° 28′ E.

From whatever quarter it is approached the aspect is singularly abrupt and picturesque. The land rises rapidly from the coast to the interior, where it forms three chains of mountains from 1800 to 2800 feet in height, intersecting the country in different directions. Except towards the summit, these are generally covered with wood, and in many parts cleft into deep ravines, through which numerous rivulets find their way to the low grounds, and terminate in about twenty small rivers, by which the whole line of coast is well watered from the foot of the mountains to the sea.

Though, from its mountainous and rugged character, a great part of the interior is not available for any useful purpose, yet extensive plains several leagues in circumference are to be found in the highlands, and in the valleys as well as along the coast, most of the ground is well adapted either for the ordinary purposes of agriculture, or for raising any description of tropical produce. Extensive forests still cover a considerable portion of the districts of Mahebourg, the Savanna, and Flacq, and in the centre of the island are several small lakes, but neither of these agencies seem to exert any material influence on the climate.

The soil in many parts is exceedingly rich, consisting either of a black vegetable mould, or a bed of stiff clay of considerable depth; occasionally the clay is found mixed with iron ore and the debris of volcanic rock. In the neighbourhood of Port Louis, and generally in the immediate vicinity of the sea, there is but a scanty covering of light friable soil over a rocky surface of coralline formation. The whole coast is surrounded by reefs of coral with the exception of a few openings through which vessels can approach the shore, and at these points the different military posts for the defence of the island have been established.

There is a marked difference in the climate of this island in different situations, the windward side enjoying a lower temperature by several degrees than the leeward, owing to the cooling influence of the south-east breeze which prevails during most of the year. The vicinity of the mountains also exerts very considerable influence on the humidity; and great varieties of temperature are experienced, according to the different degrees of elevation attained, so that at Moka and Plains Wilheims, in the high regions of the interior, fires are often necessary, when at Port Louis, though but two or three leagues distant, the heat is excessive.

As far as regards temperature, rain, physical aspect, and diversity of climate, this island exhibits a very striking resemblance to Jamaica; its latitude, too, is nearly the same, though, being to the southward of the line, the seasons are reversed, summer extending from October to April, and winter during the rest of the year. The principal rainy season is from the end of December to the beginning of April, but showers are frequent at all times, particularly in the high grounds and vicinity of the mountains.

The prevailing winds are from south-east to south, and from north-east to north. Easterly winds are rare, and usually accompanied by heavy rain; those from the west are also by no means common, and generally broken by long calms. Hurricanes are of frequent occurrence, and create great devastation, with much loss of life, but do not appear to exercise any decided influence on the health. They principally occur in January, February, and March, when, in this climate, the greatest degree of heat is combined with the greatest moisture.

So far as can be ascertained from the statistical returns of the island, the climate does not exert any prejudicial influence on the health of the white resident population, though, as we shall hereafter have occasion to show, it is by no means favourable to the negro race.

"The precise number of the white population cannot be ascertained with the same accuracy as the deaths, but the average of the years 1827 and 1832 amounted to about 13,000 females and 12,000 males, exclusive of the military and convicts, and the mortality calculated on that basis would be 1 in 41 annually of both sexes, or rather less than in Malta.

Though the data on which this estimate has been framed are not quite so precise as could be wished, yet we have every reason to believe in the accuracy of our conclusions regarding the general salubrity of the Mauritius, as it is shown in a work by M. Thomas on the statistics of the adjacent island of Bourbon, that the mortality of the same class of the population there does not exceed 1 in 45, which is nearly as low as in the United Kingdom."

*Port Louis*, the capital of the island, stands on an extensive plain about two miles in length, and nearly the same in breadth, open to the sea, but encompassed on all other sides by lofty mountains, whose bold and rocky summits, broken into a variety of peaks and chasms, present a most singular appearance.

In consequence of its position to leeward, and this rocky barrier shutting out the south-east breeze which prevails in these latitudes during a great part of the year, Port Louis is one of the hottest positions in the island. The ground in the vicinity is generally dry and rocky, but near the shore and at the head of several creeks adjacent to the harbour, as well as along the banks of a small muddy stream which flows past the town, many marshy spots are to be found, which, under a tropical sun, might be supposed likely to generate fevers of the worst type. The extensive suburbs too are generally in a very filthy state, and, during the hot and rainy season, swampy, both from situation and soil. Yet insalubrity will not be found to result.

The barracks seem to be respectable, but hot.

*Powder Mills*, a station generally occupied by a detachment. About seven miles to the north of Port Louis, in a low situation, from which the ground rises on all sides, and in the vicinity of a marshy tract of land, extending about two miles to windward. Except in that quarter, the country around the post is well cultivated, and interspersed with plantations.

*Cannonier Point*, eight miles north of this post and 15 from Port Louis, a stone casemated tower of two stories, the upper containing two, and the lower three rooms, affording sufficient accommodation for the party of from 10 to 20 men generally quartered there.

The tower stands on a low projecting spit of land, almost level with the sea; the soil is so sandy as not to admit of cultivation, and there is no marshy ground or river in the vicinity.

*Black River.*—This post is about 19 miles to the south of Port Louis, on the same side of the island, and situated on a low sandy beach at the bottom of a bay of the same name. The country in the vicinity is an uncultivated plain, interspersed with rows of date and tamarind trees; but at a considerable distance to the westward, is a range of lofty mountains which shuts out the breeze, and adds materially to the temperature.

The force consists generally of an officer and 22 men, stationed in a small barrack of three rooms, with a serjeant's quarter and guard-room at each extremity. There is also a detached building for the married soldiers, with a small hospital and other offices. All are of wood raised upon stone, and afford abundant accommodation.

The former stations are those upon the leeward side of the island. On the windward side are—

*Mahebourg.*—This station, usually the head-quarters of the corps, is situated near the head of the bay of Grand Port. The ground to the distance of three or four miles is open and tolerably well cultivated, principally under sugar cane; the soil is dry and gravelly, but on the opposite side of the bay the land swells into a ridge of high mountains extending along the coast to the north and east, and covered with forest trees. No marshes or other supposed sources of malaria exist in the vicinity, and, being open to the influence of the south-east wind, this station is reckoned one of the healthiest in the island.

The barracks consist of two buildings erected upon a low point of land, not more than 10 or 12 feet above the level of the sea, and, having a small rivulet in rear, are almost entirely surrounded by water.

The force here has seldom been under 200 men.

*Grand River Post*—is situated 14 miles to the north of Mahebourg, on the right bank of the Grand River. Behind it, a range of mountains, disposed in the form of an amphitheatre, rises to a considerable height. On the other side the country is well wooded, and in the direction of the sea there is nothing to obstruct the breeze, which blows almost constantly from the south-east. The ground is well cultivated, and no marshes or swampy situations are to be met with in the vicinity.

*Flacq.*—This post lies on the same side of the island, about 13 miles to the north of Grand River, and 21 from Port Louis by the usual road. It is open to the sea, and enjoys a refreshing breeze from the south-east during a great part of the year. The country in the vicinity is an extensive plain rising gradually towards the interior, the nearest range of mountains being seven or eight miles distant.

*Poudre d'Or*—Is another small outpost, 14 miles to the north of Flacq. The country in the vicinity is more level than at the other stations, and being farther from the mountains the climate is drier. At a short distance from the barrack is a considerable extent of marshy ground; but it does not seem to exert any prejudicial influence on the health of the troops.

To the south of Mahebourg, the previous stations being to the north of it, are Port Souillac and Jacoté.

*Port Souillac*—lies about 28 miles from Mahebourg, on the shores of a small creek or bay, at the mouth of the Savanna River. The detachment is stationed on a small headland forming the eastern side of the harbour, and about 30 feet above the level of the sea. The substratum of this headland, as well as the country adjacent, is basalt. There are no swamps or marshes in the vicinity, and the rain is speedily carried off by the natural drainage. As this post enjoys the influence of the south-east trade wind during a greater part of the year, it is cooler than most others in the island, and bears a high charaater for salubrity.

Of *Jacoté* it is not necessary to speak.

The *rations* of the white troops in this island consist of 1lb. of bread or $\frac{3}{4}$lb. of biscuit, and 1lb. of meat, fresh or salt. The proportion of salt meat is not the same at all the stations. At Port Louis and Mahebourg it was seldom issued oftener than from 10 to 14 days per month, and the proportion is now still further reduced, but at some of the smaller posts it formed the principal diet of the soldier during the period under review. As little attention is paid to the rearing of stock, and the pasturage is said to be defective, cattle are imported from Madagascar. Vegetables are very scanty, and too expensive to be used in sufficient quantities by the troops. There is no regular supper, so that the soldier passes 19 hours without food, unless he puts aside a small portion of his dinner for an evening meal.

Major Tulloch presents us with a table shewing the admissions into hospital and deaths among the white troops in the Mauritius, between the years of 1818 and 1836, inclusive. From this table we learn that among every thousand soldiers serving in the Mauritius 1249 cases of sickness have occurred in the course of the year, consequently, on an average, each man has been in hospital about once in 10 months, which is nearly the same as in the Mediterranean. The deaths which took place under medical treatment amounted to $27\frac{4}{10}$ per thousand annually; but the total mortality, including that from violent or accidental causes amounts to $30\frac{1}{2}$ per thousand. After stating the nature of these violent deaths, Major Tulloch goes on to remark, that the proportion from these causes is rather higher than in the other commands, and most of them are said to have occurred when the parties were in a state of intoxication. Besides the cases of suicide above referred to, which took place too suddenly to admit of treatment, several of the admissions recorded in the General Abstract of Diseases, arose from attempts at self-destruction, some of which ultimately proved fatal.

It will be observed that, while the mortality of the civil population little exceeds the usual ratio in the United Kingdom, that of the troops is at least twice as high as on home service. The officers also enjoy as great an exemption from sickness and mortality as any class of the resident population, so that here we may fairly conclude the sufferings of the troops to be in a great measure attributable either to their own vices and follies; or to some peculiarity in their condition, from which the others are exempt.

A marked increase, both of admissions and deaths, from almost every class of diseases, has taken place since 1828. In the six years, however, antecedent to 1818, the mortality mounted as high as 40 per thousand annually. The different stations vary materially in salubrity, the mortality being nearly one-half higher on the leeward than on the windward side of the island.

From a table it seems that the principal diseases prevail in the following order:—diseases of the stomach and bowels—abscesses and ulcers—fevers—diseases of the lungs—diseases of the liver. On comparing the diseases here with those in the Cape Town district, there is this marked difference, that in the former upwards of one-half are of so serious a character as in all probability to induce considerable injury of constitution; whereas in the latter scarcely one-third are of that description, the remainder being for the most part so trivial as not to detain the patient longer than a few days in hospital.

*Fevers.*—Though this island is situated within the tropics, there is a still greater exemption from fevers of the intermittent and remittent type than at the Cape, only 13 cases of the former and 6 of the latter having been recorded in the course of 19 years, and most of these could either be traced to causes independent of the climate, or were merely modifications of the common continued type.

The mild character of this class of diseases in the Mauritius, is sufficiently established by the circumstance of one case only in 89 having proved fatal, being a smaller proportion than in the United Kingdom, or any of the

Colonies of which the returns have yet been investigated. This may probably have arisen from many of the cases being the ephemeral result of intoxication, for which the patient was seldom more than a day or two in hospital.

The following observations must necessarily attract attention :—

" We have already drawn attention to the circumstance of this island being so similar in most respects to Jamaica, lying nearly under the same latitude, but to the south of the Line, with a temperature alike to a degree, the interior of the same bold and mountainous character, and fringed by the same succession of low lands towards the coast, with little difference in soil or moisture, and like Jamaica, intersected by numerous rivers, studded with forests, and in many parts covered with the dense vegetation of the tropics. Marshy ground is not very common in either, but that supposed agency is quite as often to be met with in the Mauritius as in Jamaica, and particularly in the neighbourhood of the capital there are many low swampy spots, sluggish streams, and receptacles of filth, which, under a tropical temperature, would appear to furnish all the elements necessary for the production of remittent fever. Yet in the Mauritius only one soldier died by that disease in the course of 19 years, out of an average force of 1606, while in Jamaica 5114 perished by it during the same period out of a force not averaging above 2578 ; thus affording a striking instance how little is known of the real causes of that fatal epidemic, and how impossible it is, even for the most diligent observer, to arrive at accurate conclusions on the subject, from information acquired merely in one portion of the globe."

*Diseases of the Lungs.*—The ratio of admissions by this class of diseases is much the same as at the Cape, but the mortality is nearly twice as high, notwithstanding the mildness of the climate and the limited range of the thermometer in this island compared with the sudden changes of temperature and violent gusts of winds so much complained of in that command.

The great source of this mortality arises from consumption, of which the proportion attacked annually has averaged 7 $\frac{7}{10}$ per thousand, being higher than in the United Kingdom, Mediterranean, or even America; although insular situations, particularly of limited extent, surrounded by a great expanse of ocean, and enjoying a mild and agreeable temperature, have generally been deemed favourable to persons predisposed to that disease. It seems to have been equally prevalent among our troops in the colony ever since our first occupation of it.

" We have frequently had occasion to remark in the course of these inquiries, that it is not always in climates where other diseases of the lungs most abound, that consumption is most prevalent. Thus in the West Indies, catarrhs and other inflammatory affections are by no means so common as in the Mediterranean or North America, yet consumption is much more so ; that disease is also found to be nearly twice as prevalent at the Mauritius as the Cape, though no such feature is manifested in others of the same class, but rather the reverse. It will also be observed that from 1818 to 1826, when all other diseases of the lungs were exceedingly rare, consumption was just as prevalent as at any subsequent period, thus indicating that its origin is not materially dependent on inflammatory action of that organ."

*Diseases of the Liver.*—This climate exhibits much the same influence in inducing hepatic affections as that of Western Africa or St. Helena, and so insidious are the attacks, that on some occasions the result of dissection exhibits total disorganization, when the feelings of the patient have led to no suspicion of the liver being affected. As in the East Indies, too, this disease is frequently combined with dysentery, though the hepatic symptoms are sometimes so obscure as to escape notice.

This class of diseases cannot be deemed altogether the result of exposure to a high temperature, since in Jamaica, where that agency is also in operation to at least an equal extent, the liver seems but little affected, and in the Mauritius during years remarkable for a very high temperature, as in 1824 for instance, they have not been more frequent than usual. Between 1812 and 1818 these diseases were extremely fatal, one in ten ending in death.

*Diseases of the Stomach and Bowels.*—This is by far the most formidable class of diseases among the troops in the Colony, more than one fourth being attacked, and upwards of 10 per thousand cut off by it annually. The great majority of the cases are reported under the head of dysentery, arising no doubt in many instances from the hepatic derangement above referred to; but, as in the West Indies, numerous cases occur also in which no such connexion can be traced. It rarely happens that the first attack of dysentery proves fatal; post-mortem examination has in almost every instance shown the tissue of the bowels to have been injured by former attacks, which rendered the sufferers particularly liable to its recurrence; the use of simple nourishing diet, with careful abstinence from all stimulants long after every dangerous symptom has disappeared, is said to be the only possible means of removing this tendency which proves so formidable a feature in the disease.

The mortality naturally falls heaviest on soldiers of advanced age, who have probably suffered from repeated attacks. Thus the youngest class of soldiers, composed principally of recruits but a short time in the colony, suffer only to one-fourth the extent of those above the age of 40.

It is singular that the officers are not affected by these diseases to a greater extent than at the Cape, or other healthy colonies; the white civil inhabitants also suffer but little from them, the mortality from all causes at a corresponding period of life not being greater than what occurs among the troops from diseases of the bowels alone.

A very marked, and not explained, increase in the frequency, if not in the mortality of diseases of the bowels has occurred since 1826. Prevailing most in the rainy months, moisture has been accused of giving rise to them; but, in Malta, they prevail in the dry months, and the wettest year known in the Mauritius, 1824, was characterised by a less than average amount of them.

*Cholera Morbus.*—Many of our readers must recollect the stress that was laid on the importation of cholera into the Mauritius by the Topaze frigate. The following statement bearing on that point may not be familiar to all.

"In 1819, when this disease prevailed to a great extent in the East Indies, it also made its appearance at Port Louis. At first it was supposed to have been imported by the *Topaze* frigate, from which several sailors were sent into the general hospital; but the certificate both by the commander and surgeon of that vessel subsequently proved, that though three cases of dysentery had terminated fatally on the voyage, no contagious disease had existed on board, nor, so far as could be ascertained, at any of the ports the vessel had sailed from, so that the appearance of this epidemic in the Mauritius remains involved in the same mystery as its origin in other Colonies."

We shall only observe of an epidemic, that with us has lost a great deal of its interest, not only because its violence but the very subject has been quite exhausted, *that*, in the Mauritius, " it exhibited nothing of a contagious character"—*that*, as in Great Britain, it did not affect all ranks equally, for among the civil population, the better classes of society suffered comparatively little, and among the officers no death took place, and only one was attacked—*that* the disease was tractable when noticed in the earlier stages, but if neglected for a few hours, collapse took place, and there was little prospect of recovery—and *that*, the proportion of deaths to admissions was considerably lower than on those recent occasions when it prevailed in the United Kingdom, Mediterranean, and America, for the deaths were :—

| | | | |
|---|---|---|---|
| In the United Kingdom .. | 1 in $3\frac{1}{4}$ | In Canada ............ | 1 in 3 |
| Gibraltar .......... | 1 ,, $3\frac{1}{2}$ | Honduras ............ | 1 ,, 3 |
| Nova Scotia ........ | 1 ,, $3\frac{1}{2}$ | the Mauritius .... ... | 1 ,, $8\frac{1}{3}$ |

*Diseases of the Brain.*—The ratio both of admissions and deaths from this class of diseases is unusually high, the former being four times, the latter twice as much so as at the Cape, indeed, the admissions occur in at least double the proportion of any other Colony. On investigation, however, we find that 393 cases reported as headache are stated to have been in most instances the result of intemperance, and 514 reported as *delirium tremens* were obviously the consequence of that vice, deducting these, the climate of the Mauritius has exerted no peculiarly unfavourable influence on this class of diseases, but rather the reverse.

In 1823 *delirium tremens* first appears in the returns. In that year four cases were reported. It progressively increased until 1835, when it had reached the number of 88. In 1836, it had sunk to 56. Intemperance, if guaged by this measure, would appear to have reached its maximum among the troops in the Mauritius. So fatal has the disease been, even among the non-commissioned officers, whose habits ought to have been such as to exempt them from its influence, that in 1835, four serjeants and one acting serjeant-major are recorded among its victims. Nor are its ultimate consequences confined to the fatal cases above recorded, for while labouring under it several have committed suicide, and numerous attempts have been made to do so, which, though unsuccessful, exposed the patient in several instances to long and protracted suffering, and ultimately led to his being permanently unfitted for the service.

Next, in point of frequency of delirium tremens to the Mauritius, comes the Windward and Leeward Command—then Bermuda—then Nova Scotia, New Brunswick, and Canada—Jamaica—the Ionian Islands—Malta—Gibraltar, and, finally, upon a par, the Cape of Good Hope and the United Kingdom.

" It says much for the salubrity of the Mauritius, that where intemperance appears so general, and its effects are manifested to so frightful an extent, the mortality among the troops should not have exceeded 3 per cent., and had those deaths been deducted which might unquestionably be traced to this vice, it would have been even as low as 2½ per cent.

With these facts before us, too, it is impossible to admit that the mortality which sweeps off so large a proportion of our troops in other tropical colonies, is mainly attributable to drunkenness, when we find that, in this island, where that vice appears at its maximum, the mortality of the troops is as low as has ever been observed in similar latitudes."

*Dropsies.*—Only nine deaths have occurred in 19 years. But, between 1812 and 1818, it was otherwise; the admissions then averaged as high as 9½, and the deaths $3\frac{9}{10}$ per thousand of the force annually. This arose principally from a species of dropsy termed *Beri Beri,* which became exceedingly prevalent among the troops in 1812, and was of so fatal a character that 41 died out of 87 attacked; so rapid was its course, that death sometimes ensued within 24 hours. In addition to the usual symptoms of dropsy, it was frequently accompanied by loss of power of the lower extremities, spasms, feeble pulse, and a state of collapse somewhat similar to cholera. The same disease has been frequently noticed in the East Indies, Ceylon, and the Burman Empire, but no case of it has ever been reported among the troops in this Colony, except in the year above referred to.

*Diseases of the Eyes* were unusually frequent in 1834.

There is nothing else on which we need stop to comment in the diseases of the Mauritius.

*Seychelles.*—A group of islands lying about 930 miles north of the Mauritius, and between 4° and 5° of south latitude. They are fifteen in number and of various extent, some so small as not to contain above 150 acres; but the principal one, named Mahé, in which the detachment is stationed, is 16 miles long and from three to four broad, with a very steep and rugged granite mountain intersecting it longitudinally; indeed, most of the islands, though resting on a bed of coral, are covered with immense masses of granite. The soil of Mahé is principally a reddish clay mixed with sand, and is watered by an abundance of small rivulets.

The weather in these islands is described as being clear, dry, and extremely equable; storms or sudden atmospherical vicissitudes are very seldom experienced, and though so near the line, the temperature being generally modified by a slight breeze, is comparatively low, and the nights are cool, with heavy dews and frequent rains. The wind is from the north-west nearly one half of the year, and from the south-east during the other half, variations being seldom experienced except when the change is taking place from one monsoon to another. There is little difference also in the seasons except during November, December, and January, when much rain falls with occasional slight squalls.

The equability of temperature may be guessed when we state that the maximum of temperature throughout the *year* was 88, and the minimum 73. We cannot, therefore, be surprised when we are told that the total population of the principal islands in the group, amounted in 1825 to 582 whites, 323 free people of colour, and 6058 slaves, all of whom are said to enjoy remarkably good health, and an exemption from the languor and debility so much experienced in other tropical climates. Extreme longevity is very common, and affections of the lungs are almost the only diseases of a serious character to which the inhabitants are subject. But it is far otherwise with the troops.

In nine years, 27 deaths have occurred out of an aggregate strength of 258, making the average ratio of mortality $10\frac{1}{2}$ per cent. annually, or nearly thrice as high as in the Mauritius.

Where the civil inhabitants are so healthy this mortality cannot altogether be attributed to the influence of climate, much of it probably originated in the diet and intemperate habits of the soldier; 11 out of the 27 deaths were from diseases of the bowels, which seem to have been materially aggravated by the constant use of salt provision, as no death has taken place from them since fresh provisions began to be issued, though previously the cases of dysentery were so severe that one in four proved fatal. The salt provisions are injurious in other ways than by mere saltness. They induce thirst, and when arrack can be had for 2s. a gallon, the soldier will not quench it with water. Intemperance is consequently carried to a still greater length than in the Mauritius, more especially as the officers in command have no means of carrying into effect the punishments likely to restrain it.

Diseases of the liver have also been exceedingly fatal among this detachment, the deaths from them alone having averaged nearly 3 per cent. of the force annually, and as at the Mauritius, a large proportion of the dysenteric affections may perhaps be attributed to derangement of that organ. It will be observed that two fatal cases of consumption occurred among the small force stationed here, a sufficient proof that even in the most equable of climates, that disease carries off as large a proportion of victims as in the most inclement regions; the natives, too, are said to suffer considerably from it, but we possess no details to show precisely to what extent.

*Black Pioneers.*—These military labourers have been enlisted for the purpose of relieving the European soldiers from the performance of fatigue and other duties, which subjected them to much exposure. They are all negroes who have either been born in the Mauritius, or brought from Madagascar and Mozambique on the Eastern Coast of Africa. They are described as being a more robust and athletic race than those composing the West India regiments.

A table exhibits the admissions into hospital and deaths among these troops since 1825. As regards both, the ratio is almost exactly the same as among the black troops and pioneers in the Windward and Leeward Command, the former being as 839 to 820, the latter as 37 to 40 per thousand of mean strength annually, so that the Mauritius and the West Indies seem alike unsuited to the constitution of the negro. This shows how vain is the expectation, even under the most favourable circumstances, of that race ever keeping up or perpetuating their number in either of these colonies, when men in the prime of life, selected for their strength and capability for labor, subject to no physical defect at enlistment, and secured by military regulations from all harsh treatment, die nearly four times as rapidly as the aboriginal inhabitants of the Cape, or other healthy countries, at the same age, and at least thrice as rapidly as the white population of the Mauritius.

Indeed, so fast is the negro race decreasing there, that in five years the deaths have exceeded the births by upwards of 6000 in a population of 60,000.

However difficult it may be to assign an efficient cause, it is certain that the inhabitants of different countries have different susceptibilities for particular diseases. Fevers, for instance, have little influence on the negro race in the Mauritius, for no death has occurred from them, and the admissions have been in much the same proportion as among an equal number of persons in the United Kingdom; but here, as in all other Colonies in which we have been able to trace the fatal diseases of the negro, the great source of mortality has been those of the lungs, indeed, more die from that class alone than of Hottentot troops at the Cape from all diseases together; but the latter are serving in their natural climate, the former in one to which their constitution never has adapted, and probably never will adapt itself.

Major Tulloch compares the mortality of the negro from diseases of the lungs in various colonies. There died annually of these affections, per 1000 of mean strength—

| | |
|---|---|
| West Coast of Africa .. .. .. .. | $6\frac{3}{10}$ |
| Honduras .. . .. .. .. .. | $8\frac{1}{10}$ |
| Bahamas . .. .. .. .. .. .. | $9\frac{7}{10}$ |
| Jamaica .. .. .. .. .. .. | $10\frac{3}{10}$ |
| Mauritius .. .. .. .. .. .. .. | $12\frac{9}{10}$ |
| Windward and Leeward Command.. | $16\frac{5}{10}$ |
| Gibraltar .. .. .. .. .. .. .. | $33\frac{5}{10}$ |

"Thus in his native country the negro appears to suffer from these diseases in much the same proportion as British troops in their native country, but so soon as he goes beyond it, the mortality increases till, in some colonies, it attains to such a height as seemingly to preclude the possibility of his race ever forming a healthy or increasing population.

It is in vain that we look for the cause of this remarkable difference, either in temperature, moisture, or any of those appreciable atmospheric agencies, by which the human frame is likely to be affected in some climates more than in others, and it is consequently impossible, from any other data than that which the experience of medical records furnishes, to say where this class of troops can be employed with advantage. Nearly two-thirds of the mortality from diseases of the lungs among negroes, arises from pulmonary consumption; and it is worthy of remark, as showing how little that disease affects the natives of some tropical climates, though it proves so fatal to those of others, that among 71,850 native troops serving in the Madras Presidency, the deaths by every description of disease of the lungs did not, on the average of five years, exceed 1 per thousand of the strength annually."

It is rather curious that the Black Pioneers suffer more from hepatic disease than the white troops, the mortality being as $5\frac{7}{10}$ to 4 per thousand of the strength. This is not the case either in Africa or the West Indies.

The diseases of the stomach and bowels are not half so frequent or so fatal among the black troops as the white, indeed, they do not suffer more from them than when serving on the coast of their native continent. Even that proportion, however, is a sufficiently formidable item in the mortality, and one which in itself would prove a serious obstacle to the increase of any population equally subject to it. Here, as in the West Indies, rheumatic affections are nearly twice as common as among the white troops.

There is a *section* on the :—

*Influence of Age and Length of Residence on the Mortality among Troops serving in the Mauritius.*

Some tables are presented to us, which shew that the deterioration of constitution with the advance of age in the Mauritius must have been extremely rapid. On comparing the progressive increase of mortality at the same ages among the Dragoon Guards and Dragoons serving in the United Kingdom, it appears that though, between the ages of 18 and 25, the ratio is but 7 per thousand higher than in this country, that difference increases, between the ages of 40 and 50, to nearly 60 per thousand. Consequently residence in this colony seems to affect the oldest at least eight times as much as the youngest class of soldiers.

"It is out of the question to attribute this rapid deterioration of life entirely to the influence of climate, because in that case we should find the same feature manifested in a corresponding degree among the higher ranks of officers, who, though between the ages of 40 and 50, have suffered comparatively little in this Colony; and so far from any similar feature being manifested among the civil population, extreme longevity is nearly as common as in this kingdom."

Major Tulloch naturally attributes this constitutional deterioration to intemperance. That the latter plays a very efficient part in the production of it, we do not for an instant doubt.

"Whether this rapid deterioration of life is the result of the soldier's own intemperance or of the influence of climate, however, it seems equally necessary, as a remedial measure, to limit as much as possible the period of his residence in this island, as has recently been done in the West Indies, by causing the latter years of his tour of foreign service to be passed in some other colony where the facilities to intemperance are fewer and the climate more favourable to a broken constitution. This measure certainly offers a greater probability of reformation than leaving the soldier for a long series of years exposed to the temptations which originally seduced him into a course of dissipation, and might afford some hope of such a renovation of constitution as would render him for many years a healthy and efficient soldier, instead of sinking into an untimely grave or being thrown at a comparatively early age on the pension list.

To such an arrangement there might be an objection if the health of the soldier was found to improve by length of residence, or, as it is technically called, by acclimatization; but the fallacy of such a supposition is not only proved indirectly from the facts before stated in regard to the increase of mortality with the advance of age, but directly by reference to the relative proportion of deaths during each successive year of residence, among the different corps which have arrived in the island since 1826."

*Mortality of Officers.*—The extreme rarity of deaths among officers in this Colony has long been a subject of remark, indeed the proportion from disease is as low as in the United Kingdom, a sufficient proof that, though within the tropics, the climate of this island, like that of St. Helena, does not of itself exercise any decidedly unfavourable influence on the health of Europeans. Officers exhibit none of that tendency to diseases of the bowels which characterises the troops.

Such are the main facts and features of the voluminous Report before us. Its author observes, on concluding it:—"when the sphere of our observations shall have been extended over all the foreign possessions of the British Crown, as will shortly be the case, a series of deductions will be obtained in regard to the influence of the same diseases on similar bodies of troops in almost every quarter of the globe, sufficiently ample to afford the means not only of testing the accuracy of our previous conclusions, but of extending them much farther than has hitherto been deemed prudent."

For this very reason we forbear indulging in any observations of our own. When the final reports and deductions are before us, it will be time enough to apply the individual experience of the medical man to the generalizations of statistics. We will content ourselves at present with remarking on the general ability displayed by Major Tulloch, and on the absence from prejudice and bias which he shews. Perhaps this very candour and impartiality may be carried rather far, and breed a degree of scepticism unfavourable rather than favourable to the discovery of truth, in sciences which do not admit of demonstration. Be this as it may, it is a fault on the right side, more particularly on subjects where speculation has hitherto been rampant, and assertion vaunted as truth.

We would earnestly direct the attention of our readers to these Reports. We repeat that they do not merely concern our troops and colonies. They affect the very primary doctrines of medicine, and they materially affect, or should affect, our practice. They have given the death-blow to the expatriation of invalids affected with pulmonary alterations. They serve also to shew us the salubrity of our calumniated climate, and to lower our aspirations for that "sweet South," whose sunny skies and luxuriant plains too commonly smile but to betray. Statistics dispel those illusions of poesy, and even prove that consumption, the reproach of our fickle seasons, lurks as fatally in the balmy Italian Zephyr, or the sultry tropical breeze.

